# Therapeutic efficacy of cancer stem cell-based vaccine in colorectal murine model: reduced tumor growth and prolonged survival

**DOI:** 10.1186/s12885-026-15925-3

**Published:** 2026-03-29

**Authors:** Farideh Hashemi, Masoumeh Dehghan Manshadi, Sadegh Safaei, Hossein Aminianfar, Mahmood Bozorgmehr, Leila Eini, Ahmad Shariftabrizi, Mahdieh Razmi, Marzieh Naseri, Roya Ghods, Zahra Madjd

**Affiliations:** 1 Oncopathology Research Center, Iran University of Medical Sciences, Tehran, Iran; 2 Department of Molecular Medicine, Faculty of Advanced Technologies in Medicine, Iran University of Medical Sciences, Tehran, Iran; 3 Department of Pathology, Faculty of Veterinary Medicine, University of Tehran, Tehran, Iran; 4 Flow Cytometry Core Facility (CF-FLOW), Helmholtz Zentrum München – German Research Center for Environmental Health, Munich, Germany; 5 Department of Veterinary Basic Sciences, Islamic Azad University, Tehran, SR.C Iran; 6 Division of Nuclear Medicine, Department of Radiology, University of Iowa Carver College of Medicine, Iowa City, IA USA; 7 Department of Developmental, Molecular, and Chemical Biology, Tufts University School of Medicine, Boston, MA USA

**Keywords:** Cancer stem cells (CSCs), Therapeutic CSC-based vaccine, Colorectal murine model, Tumor growth, Survival rates

## Abstract

**Background:**

Various forms of cancer immunotherapy are promising in overcoming the obstacles posed by resistance to conventional chemo- and radiotherapy. Cancer vaccines could serve as beneficial adjuncts to conventional therapies, offering the potential for fine-tuning to reduce relapse and related mortality. Continuing prior investigations, a therapeutic colorectal cancer stem cell (CSC)-based vaccine was developed to explore whether this vaccination could inhibit the formation and prolong survival rates in a mouse model of colorectal cancer.

**Methods:**

CSCs were enriched from the CT-26 cell line using sphere formation assay and characterized by real-time q-PCR for stemness genes (*Oct4*,* Sox2*, and *Nanog*) and tumorigenesis assay in syngeneic BALB/c mice. Different groups of mice were intraperitoneally immunized with the CSC lysate-based vaccine, the parental cell lysate-based vaccine, and control groups following subcutaneous challenge with CT-26 cells. Beyond analyzing tumor growth and survival rates, histological analysis of tumor tissues was conducted using comprehensive hematoxylin and eosin (H&E) staining, and antibody responses in vaccinated mice were evaluated by flow cytometry and immunofluorescence.

**Results:**

Immunization of tumor-bearing mice with the CT-26 CSC lysate-based vaccine caused delayed tumor formation, reduced tumor growth rate, and enhanced survival rate compared to the control groups. The histological responses observed in the lysate vaccination subgroups indicated a potent immune response. Furthermore, flow cytometry and immunofluorescence analyses demonstrated the production of anti-CSC and anti-parental cell antibodies in mice immunized with CT-26 CSC and parental cell lysates.

**Conclusion:**

These findings suggest that targeting CSCs using a CSC lysate-based vaccine can stimulate cellular and humoral immunity and represent a novel therapeutic approach to complement conventional antitumor therapies.

**Graphical abstract:**

**Figure:**
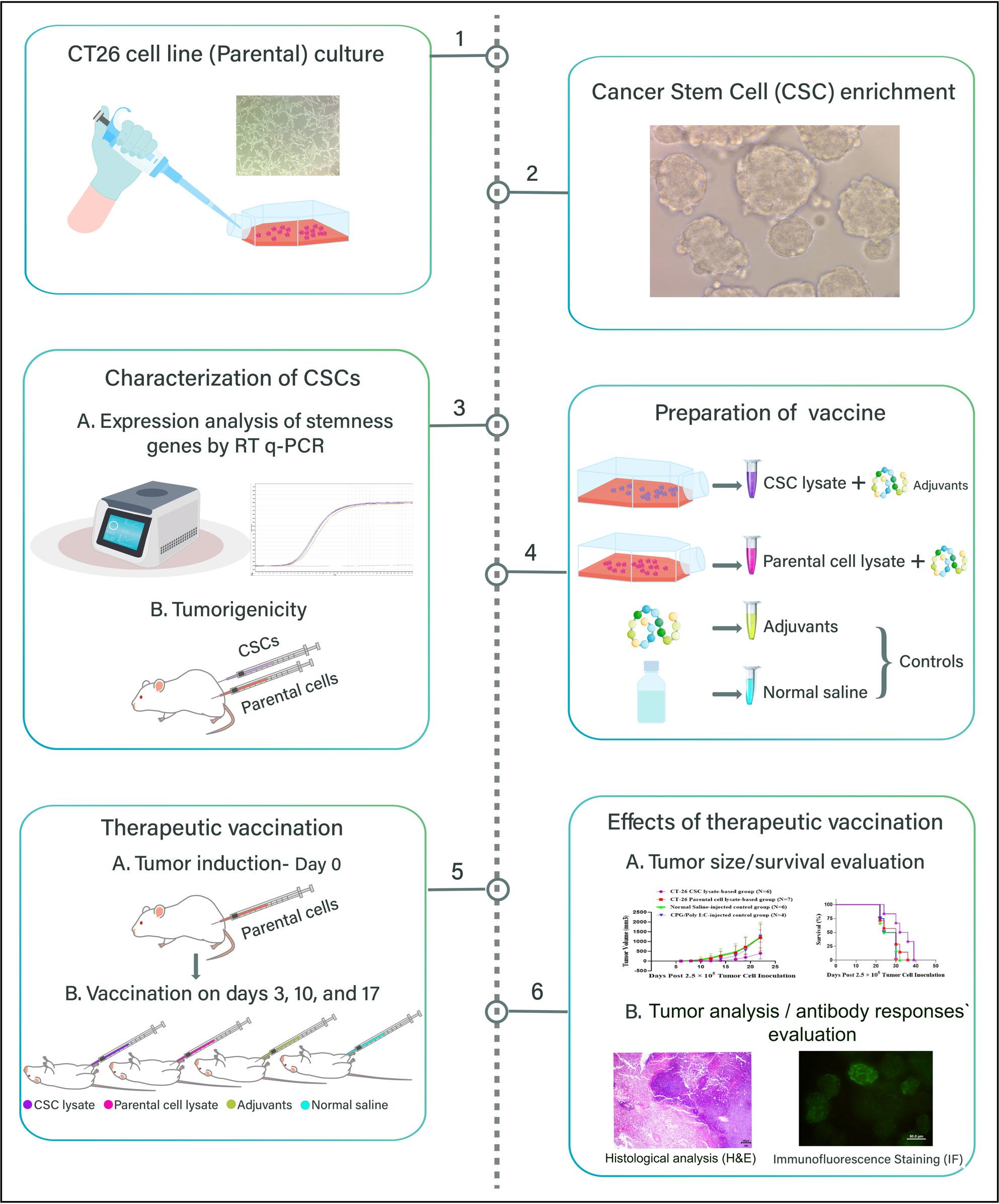
An overview of the experimental design. Cancer stem cells (CSCs) were enriched from the CT-26 cell line and characterized using real-time q-PCR for stemness genes and tumorigenesis assay in BALB/c mice. Following a subcutaneous CT-26 cell challenge, mice were injected intraperitoneally with the CSC lysate-based vaccine, parental cell lysate vaccine, and normal saline and adjuvant (control groups), with a 7-day interval between each injection. Beyond analyzing tumor growth and survival rates, histological analysis of tumor tissues was performed using H&E staining, and antibody responses in vaccinated mice were assessed by flow cytometry and immunofluorescence

**Supplementary Information:**

The online version contains supplementary material available at 10.1186/s12885-026-15925-3.

## Significance statement

The findings of this study suggest that a therapeutic colorectal cancer stem cell (CSC) lysate-based vaccine can delay tumor progression, improve survival outcomes, and stimulate immune responses in a mouse model, supporting its potential role as a complementary immunotherapeutic approach to conventional cancer treatments.

## Introduction

Colorectal cancer (CRC) is the third most frequently diagnosed cancer and the second leading cause of cancer-related mortality worldwide, posing a major threat to human health [1]. Even with advances in surgery, chemotherapy, and targeted therapies, CRC is one of the most difficult malignancies to treat due to the high frequency of recurrence and metastasis and frequent resistance to first- and second-line therapies [2–6]. Although the cause of relapse and metastasis has not been completely elucidated, it is hypothesized that the presence of a small subset of self-renewing cancer cells, known as cancer stem cells (CSCs), within the tumor mass is a major contributing factor to tumor recurrence and metastasis [7–10]. In contrast to differentiated cancer cells, CSCs remain quiescent during the cell cycle, rendering them less susceptible to conventional cytotoxic drugs specifically designed to attack rapidly dividing cells [2, 11, 12]. CSCs are a unique subset of tumor cells characterized by three key properties: multidrug resistance, ability to evade cell death (apoptosis), and capacity to manipulate the tumor’s surrounding environment, rendering them exciting targets for potential cancer treatment [13–16]. Hence, selectively targeting and eliminating CSCs is important in overcoming chemoresistance and improving prognosis.

Until now, different approaches, including chimeric antigen receptor-modified T cells (CAR-T cells) against CSC antigens, monoclonal antibodies targeting CSC surface molecules, energy metabolic enzyme inhibitors, signal pathway inhibitors, and differentiation induction, have been used to inhibit CSC function or reduce CSC frequency [17–22]. While there are numerous approaches for eliminating CSCs, the primary focus of the prior research has been on targeting tumor-associated antigens (TAA) as opposed to the tumor-specific antigens (TSA) [23] and the absence of effective tumor-specific targets remains a critical issue in this regard [23]. Hence, recognizing neoantigens that are crucial in the self-renewal of CSCs might potentially enhance the identification of interesting targets for effective immunotherapy [24].

Although identifying particular CSC neoantigens remains difficult [24], whole CSC-based vaccination strategies have been employed to address this challenge [22, 25–27]. A whole CSC-based vaccine can introduce comprehensive antigens that would help in their being recognized by the immune system and activate anti-CSC immunity, leading to the targeting of CSCs and tumor cells and ultimately decreasing the likelihood of cancer relapse and metastasis [22, 25, 28, 29]. In fact, the utilization of whole CSCs in vaccination appears to be more effective in cancer therapy and also would help in the identification of specific antigens that can induce strong immune responses against tumor cells [25, 29].

In our previous work, we investigated the efficacy of a prophylactic CSC-based vaccination strategy using a syngeneic CT-26 colorectal cancer model. That study demonstrated that vaccination prior to tumor challenge inhibited subcutaneous tumor growth, induced a humoral immune response, and significantly prolonged survival in vaccinated mice [30].

However, prophylactic and therapeutic cancer vaccines address fundamentally different biological contexts [31]. While prophylactic vaccination is administered in the absence of an established tumor, therapeutic vaccination must elicit effective antitumor immunity in tumor-bearing hosts [31]. Consequently, the therapeutic efficacy of a CSC-based vaccine cannot be inferred from prophylactic models and must be evaluated independently in clinically relevant settings.

The present study aimed to expand our prior research and evaluate the efficacy of a therapeutic CSC-based vaccine using CSC-enriched cell lysate derived from the CT-26 CRC cell line on tumor growth, mouse lifespan, and antibody production in a BALB/c mouse model of colorectal cancer. We comprehensively evaluated and compared the efficacy of two different sources of antigens derived from the CT-26 cell line and CT-26 spheroid cells on tumor volume and survival rate in an immunocompetent mouse cancer model.

## Materials and methods

### Phase I: CSC enrichment using sphere formation assay and confirmation of stem cell characteristics

#### CT-26 cell line culture

The CT-26 murine colorectal cancer cell line was purchased from the National Cell Bank of Iran (NCBI, Tehran, Iran). Cells were cultured at 37 °C in a 5% CO2 atmosphere in complete media consisting of Dulbecco modified Eagle medium/F12 (DMEM/F12) (Gibco, Germany), 100 U/mL penicillin, 100 µg/mL streptomycin (Biowest, France), and 10% fetal bovine serum (FBS) (Gibco, Germany). When CT-26 cells reached 70–90% confluence, cells were harvested enzymatically using trypsin/EDTA (0.05%) (Gibco, Germany) and passaged.

#### CT-26 CSC enrichment culture (spheroid cell culture)

To obtain CT-26-derived spheres, 1 × 10^5^ murine CT-26 cells in a logarithmic growth phase (from second or third passages) were seeded into 1.2% poly-HEMA (Sigma, USA) coated dishes in serum-free DMEM/F12 (Gibco, Germany) supplemented with 1% non-essential amino acid, 1% penicillin-streptomycin, 2 mM L-glutamine, 20 ng/mL epidermal growth factor (EGF) (PeproTech, USA), 10 ng/mL fibroblast growth factor basic (bFGF) (PeproTech, USA), and 2% B27 supplement (Gibco, Germany). After 5–7 days of incubation, CT-26 CSC-enriched spheroids were collected and dissociated into a single-cell suspension using trypsin/EDTA, and cell viability was assessed by the Trypan Blue exclusion assay. Third-generation spheroids were used for subsequent experiments.

#### Scanning Electron Microscopy (SEM) imaging of spheroid cell morphology and structure

Spheroid cells were washed two times with PBS and fixed in 2.5% glutaraldehyde (Sigma-Aldrich) for 20 min at 4 °C. After fixation, cells were immediately subjected to gradient dehydration using 30%, 50%, 70%, 80%, and twice with 100% ethanol (Sigma-Aldrich) for 3 min at each concentration. Following air drying at room temperature for 15–20 min, samples were gold-coated and observed using a scanning electron microscope (Seron Technology, AIS-2100, Korea).

#### Characterization of spheroid cells

##### mRNA expression analysis of stemness genes (*Oct4*,* Sox2*,* and Nanog*) by real-time q-PCR

To evaluate the mRNA expression of stemness genes, including *Oct4*,* Sox2*, and *Nanog*, total RNAs were isolated from the samples (RNeasy Mini Kit (GeneAll, Korea)). Then, Nanodrop (ThermoFisher Scientific, USA) and agarose gel electrophoresis were applied to evaluate RNA quantity and purity. After removing genomic DNA contamination by DNase I, 2 µL of total RNA and cDNA synthesis kit (Yektatajhize, Iran) were used for the reverse transcription (RT) reactions for each sample. Real-time PCR was performed on 1 µL of cDNA using PCR Master Mix Green-High Rox A325402-25 (Ampliqon, Denmark) on the Rotor-Gene Q Light Cycler (Qiagen, Germany). All reactions were performed in duplicate. In brief, PCR amplification was carried out using an initial denaturation at 95 °C for 15 min, followed by 40 cycles for 15 s at 95 °C, 15 s at 60 °C, and 15 s at 72 °C. The results were analyzed using the 2^−ΔΔCt^ relative quantification method, and the expression of glyceraldehyde-3-phosphate dehydrogenase (Gapdh) was used as an internal control. The primer sequences are provided in Table 1. RT-qPCR data are presented as individual biological replicates overlaid with mean values to accurately reflect data dispersion.

**Table 1:** RT-qPCR Primers and expected product sizes

Gene of interest	Sequence	PCR Product Size (bp)
*Oct4*	*F 5’- GTTCTCTTTGGAAAGGTGTTC − 3’* *R 5’- GCATATCTCCTGAAGGTTCTC − 3’*	*147*
*Sox2*	*F 5’- AAAGGGTTCTTGCTGGGTTT − 3’* *R 5’- AGACCACGAAAACGGTCTTG − 3’*	*151*
*Nanog*	*F 5’-TGATTTGGTTGGTGTCTTG − 3’* *R 5’-TGTGATGGCGAGGGAAG − 3’*	*176*
*Gapdh*	*F 5’- AACTTTGGCATTGTGGAAGG − 3’* *R 5’- CACATTGGGGGTAGGAACAC − 3’*	*222*

##### Animals and ethical statement

Female BALB/c mice aged 6–8 weeks were purchased from the Royan Institute (Tehran, Iran). All the mice were maintained in standard conditions at the Iran University of Medical Sciences (IUMS) animal facilities. All animal work was performed according to the committee guidelines and protocols approved by the animal research committee of IUMS (Ethics Committee Number: IR.IUMS.REC.1398.954). This study has been reported in accordance with the ARRIVE guidelines 2.0.

##### In vivo tumorigenicity

To compare the tumor induction potency of the CT-26 spheroid cells and the CT-26 parental cells, an equal number of both cells (2 × 104 and 1 × 104 cells) were injected subcutaneously into the opposite flanks of the same BALB/c mice (n = 3 mice in each group). The volume of the tumor was monitored by measuring two perpendicular tumor diameters using calipers every three days by a blind and experienced person to avoid bias in data collection. Tumor size was compared between the two groups, and when tumors reached a maximum length of 20 mm in one dimension, mice were euthanized. Animal anesthesia was administered using a combination of xylazine and ketamine, and following anesthesia, euthanasia was carried out using spinal cord injury (SCI). Tumor tissues were removed and processed for paraffin section preparation. Following 10% buffered formalin fixation and dehydration, paraffin-embedded blocks were sectioned at 5 μm for hematoxylin and eosin (H&E) staining, and images were acquired using a light microscope (Nikon, Japan).

### Phase II: vaccination

#### Preparation of cell lysate

CT-26 cell line (parental cells) and CT-26 spheroid cell lysates were prepared as previously described [30]. Briefly, the CT-26 parental cells and CT-26 spheroids in the third passage were exposed to 0.1 mM 2-mercaptoethanol (2-ME) for 16 h before harvesting to induce cellular stress prior to lysis. This pretreatment was applied based on evidence that stress induction before tumor cell lysis can enhance the immunogenic properties of tumor cell lysates [25]. The harvested cells were washed five times with PBS buffer, treated with a protease inhibitor, and subsequently lysed by ten cycles of repetitive rapid freezing in liquid nitrogen and thawing in a 37 °C water bath. The disrupted cells were centrifuged at 20,000 ×g for 20 min at 4 °C. The supernatant was then collected, and the protein concentration was determined using the bicinchoninic acid (BCA) assay according to the manufacturer’s instructions (TaKaRa, Japan). The lysates were subsequently stored at − 80 °C until further use.

#### Efficacy evaluation of therapeutic vaccination in mouse model

The colorectal cancer model was established by subcutaneously injecting 5 × 10^5^ (Group A) and 2.5 × 10^5^ (Group B) CT-26 parental cells in 0.1 mL of PBS into the right flank of BALB/c mice on day 0 using a 27-gauge needle. The tumor-inoculation dose of 5 × 10⁵ cells was chosen to maintain continuity with our previous prophylactic vaccination study and to enable direct comparison between prophylactic and therapeutic vaccination settings using the same CSC-based vaccine platform. A lower inoculation dose (2.5 × 10⁵ cells) was included as an additional experimental condition to assess vaccine efficacy under reduced tumor burden, given the influence of tumor cell inoculum size on tumor latency, growth kinetics, and immune responsiveness. After 3 days of tumor inoculation, to assess the therapeutic effect of CSC lysate vaccination, female BALB/c mice in each group were randomly divided into four subgroups that comprised the CT-26 CSC lysate-based vaccine, the CT-26 parental cell lysate-based vaccine, the CpG/Poly (I: C)-injected control group, and a normal saline-injected control group (four to seven mice per group). The mice were vaccinated intraperitoneally (IP) three times with 50 µg of prepared cell lysate and adjuvant per inoculation, with a 7-day interval between vaccinations. Three µg Phosphorothioate-modified CpG oligodeoxynucleotide (Class B CpG oligonucleotide, InvivoGen-ODN 1826) and 25 µg Polyinosinic-Polycytidylic acid (Poly (I: C), SIGMA, P1530) were used as adjuvants and were added to each vaccine dose. Injection volumes and vaccination schedules were identical across all experimental groups. In the CpG/Poly(I: C) control group, animals received the same doses of CpG and Poly(I: C) as those used in the vaccine formulations but without lysate antigens, whereas the saline control group received the same injection volume without adjuvants or lysate. The mice in the CpG/Poly (I: C)-injected control group and the normal saline-injected control group were injected IP with CpG/Poly (I: C) and 0.1 ml of normal saline, respectively.

#### Tumor size and survival evaluation

Every two days, the volume of the tumors was determined by measuring two perpendicular tumor diameters using a caliper. To avoid bias, this assignment was carried out by an experienced individual blinded to the group assignments. As described previously, tumor volume measurement was calculated using the following formula: tumor volume (mm^3^) = (length × width^2^) ÷ 2, where width was the shorter dimension [30]. An overall assessment of the mice’s health was evaluated and followed for general health conditions, diet, and behavior during the study. The endpoint for the experiments was a tumor diameter reaching 20 mm in one dimension when the mice were sacrificed. Animal anesthesia was administered using a combination of xylazine and ketamine, and following anesthesia, euthanasia was carried out using spinal cord injury (SCI). For the survival analysis, the time of death was recorded for mice in each group to calculate the survival rate.

### Phase III: tumor tissue analysis and immune response evaluation

#### Histological analysis of tumor tissues

Tumor tissues were harvested after euthanasia, fixed in 10% neutral buffered formalin for 24–48 h, paraffin-embedded, and sectioned at 5 μm thickness. Sections were stained with hematoxylin and eosin (H&E) using standard protocols and examined under a light microscope by an experienced pathologist (H.A.F.). Quantitative histological assessments included mitotic index, necrotic area, and fibrosis score. The mitotic index was determined by counting mitotic figures in 10 consecutive high-power fields (HPF, 400×) within the most proliferative tumor regions, and the number of mitotic figures per HPF was normalized to the total number of tumor cells counted in the same fields and expressed as a percentage. Necrotic areas were quantified from digital images captured at 100× magnification using Axiovision Rel. 4.8 software, where necrotic regions were delineated and calculated as: Necrotic Area (%) = (Total necrotic area/Total tumor area) ×100. Fibrosis was assessed semi-quantitatively based on the extent of fibrous tissue distribution and scored as 0 (none), 1 (mild, < 25%), 2 (moderate, 25–50%), or 3 (severe, > 50% of tumor area). All analyses were performed in a blinded manner, and statistical comparisons were conducted using one-way ANOVA, with *p* < 0.05 considered statistically significant.

#### Antibody responses’ evaluation

The antibody responses against CSCs and parental cells in vaccinated mice were assessed by flow cytometry analysis and immunofluorescence staining (IF). Equal volumes of serum from immunized mice were initially pooled in each group to perform these tests. This approach was intentionally adopted because the objective of the experiment was to compare antibody-mediated immunoreactivity between experimental groups rather than to evaluate inter-individual variability within the same group. Accordingly, pooled sera were used as a representative measure of the group-level humoral immune response, as commonly applied in immunological studies aimed at detecting and comparing antigen-specific antibody reactivity between groups [32, 33]. Therefore, the flow cytometry data reflect group-level measurements.

##### Antibody responses’ evaluation by flow cytometry

Single-cell suspensions were prepared from spheres and parental cells using trypsin-EDTA. All cell populations were incubated with a 1:50 dilution of pooled vaccinated mice sera, followed by incubation with a secondary antibody, FITC-conjugated sheep anti-mouse IgG (Avicenna Research Institute (ARI), Tehran, Iran), diluted 1:100. Unstained cells were included to determine intrinsic cellular autofluorescence and to establish gating thresholds. Additionally, cells incubated with serum from normal (non-immunized) mice at the same dilution and stained using the same secondary antibody served as negative (isotype-equivalent) controls to rule out nonspecific binding. At least 10,000 events were acquired for each sample using a flow cytometer (Thermo Fisher Scientific, USA), and data were analyzed using FlowJo software (V10). All samples were processed under identical experimental and acquisition settings.

##### Antibody responses’ evaluation by immunofluorescence staining (IF)

Parental and sphere cells were fixed with 4% paraformaldehyde at room temperature (RT) for 30 min, exposed to 96% and 70% ethanol, and washed with PBS. Then, the cells were permeabilized with 0.3% Triton X-100 and washed again. Subsequently, all cell populations were washed twice with PBS containing 0.5% BSA for 5 min, then incubated with 5% normal sheep serum and 2.5% BSA in PBS for 30 min at room temperature to block the nonspecific binding, followed by three PBS washings. The CT-26 spheroid and parental cells were incubated with a 1:50 dilution of pooled mouse sera from each group for 90 min. The same normal (non-immune) mouse serum dilution was used as a control. The cells were then incubated with FITC-conjugated sheep anti-mouse IgG (Avicenna Research Institute (ARI), Tehran, Iran), diluted 1:100, at RT for an additional 45 min before being washed three times with PBS, as described previously [30]. The nuclei were stained with 0.5 µg/mL DAPI (4’, 6-diamidino-2-phenylindole; Sigma-Aldrich, Germany). After staining, samples were evaluated using fluorescence microscopy (Olympus IX71; 460 nm DAPI filter). IF staining was used for qualitative visualization of antibody reactivity, whereas the percentage of positively stained cells was quantitatively assessed by flow cytometry.

### Statistical analysis

Statistical comparisons were conducted using Student’s t-test. If *p* ≤ 0.05, differences were considered statistically significant. The Mann-Whitney U test, a two-tailed, nonparametric probability test, was used to compare the volumes of tumors in vaccinated and control groups. The two-sided log-rank (Mantel-Cox) test was utilized to analyze the survival analysis. GraphPad Prism version 8.0 (GraphPad Software, La Jolla, CA, USA, www.graphpad.com/) was used for data analysis. As no observations were censored during follow-up, censoring tick marks are not displayed on the Kaplan–Meier curves.

## Results

### Phase I: CSC enrichment using sphere formation assay and confirmation of stem cell characteristics

#### CT-26 CSC enrichment using sphere formation assay

**Fig. 1:**
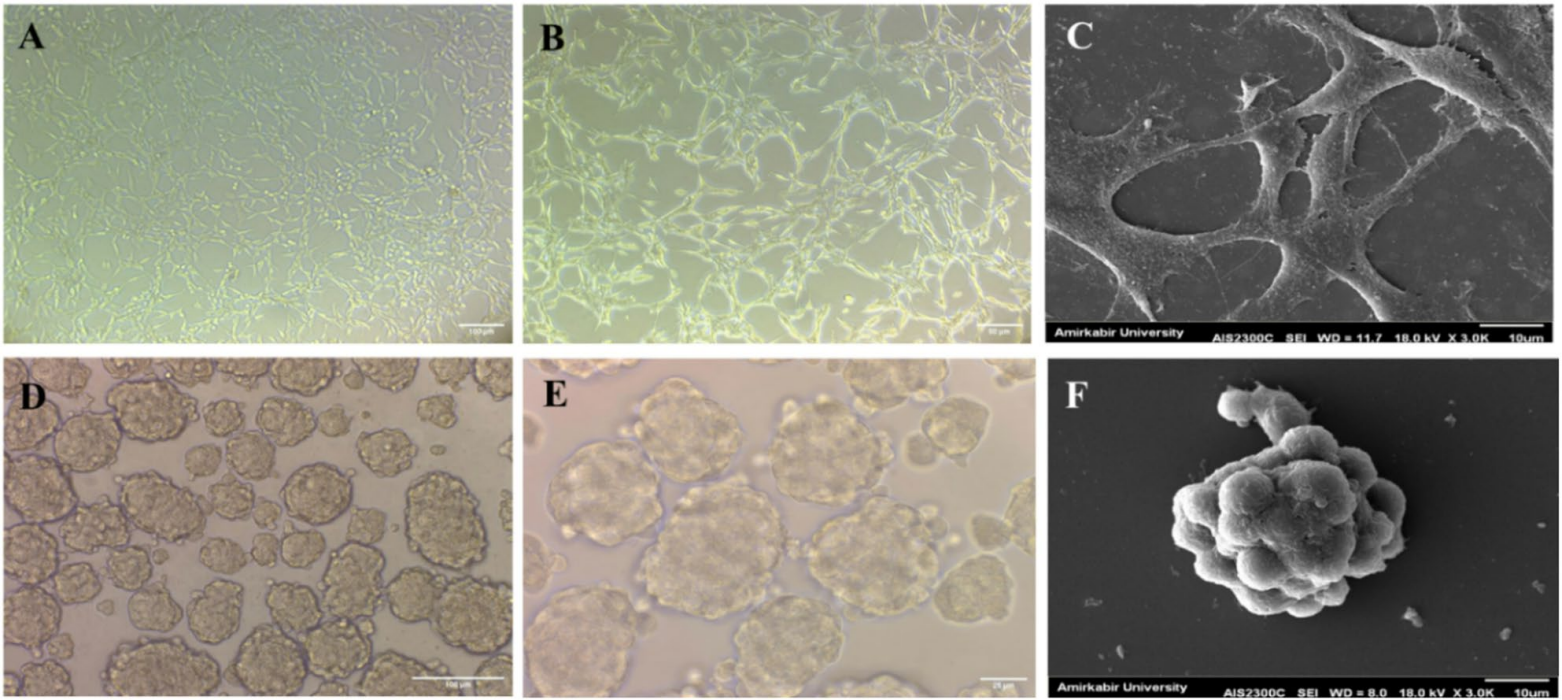
Morphology of CT-26 parental and spheroid cells. **A**,** B**, CT-26 parental cells cultured under 2D conditions; magnifications ×100 and ×200. **C**, SEM image showing the morphological features of CT-26 parental cells (scale bar = 10 μm). **D**,** E**, CT-26 spheroid cells derived from CT-26 parental cells and cultured in spheroid culture medium; magnifications ×200 and ×400. **F**, SEM image showing the structural characteristics of CT-26 spheroid cells (scale bar = 10 μm)

The CT-26 cell line (parental cell) was used to generate colorectal cancer spheroids under serum-free and non-adherent conditions (Figs. 1A–F). SEM imaging was performed to evaluate the morphological characteristics of parental cell and spheroids derived from the CT-26 cell line regarding their surface features (Fig. 1C and F). Spheroids exhibited a round morphology and compact structure (Fig. 1F).

#### Characterization of the CT-26 spheroid cells

##### Higher expression of stemness genes in spheroid cells relative to their parental cells

**Fig. 2:**
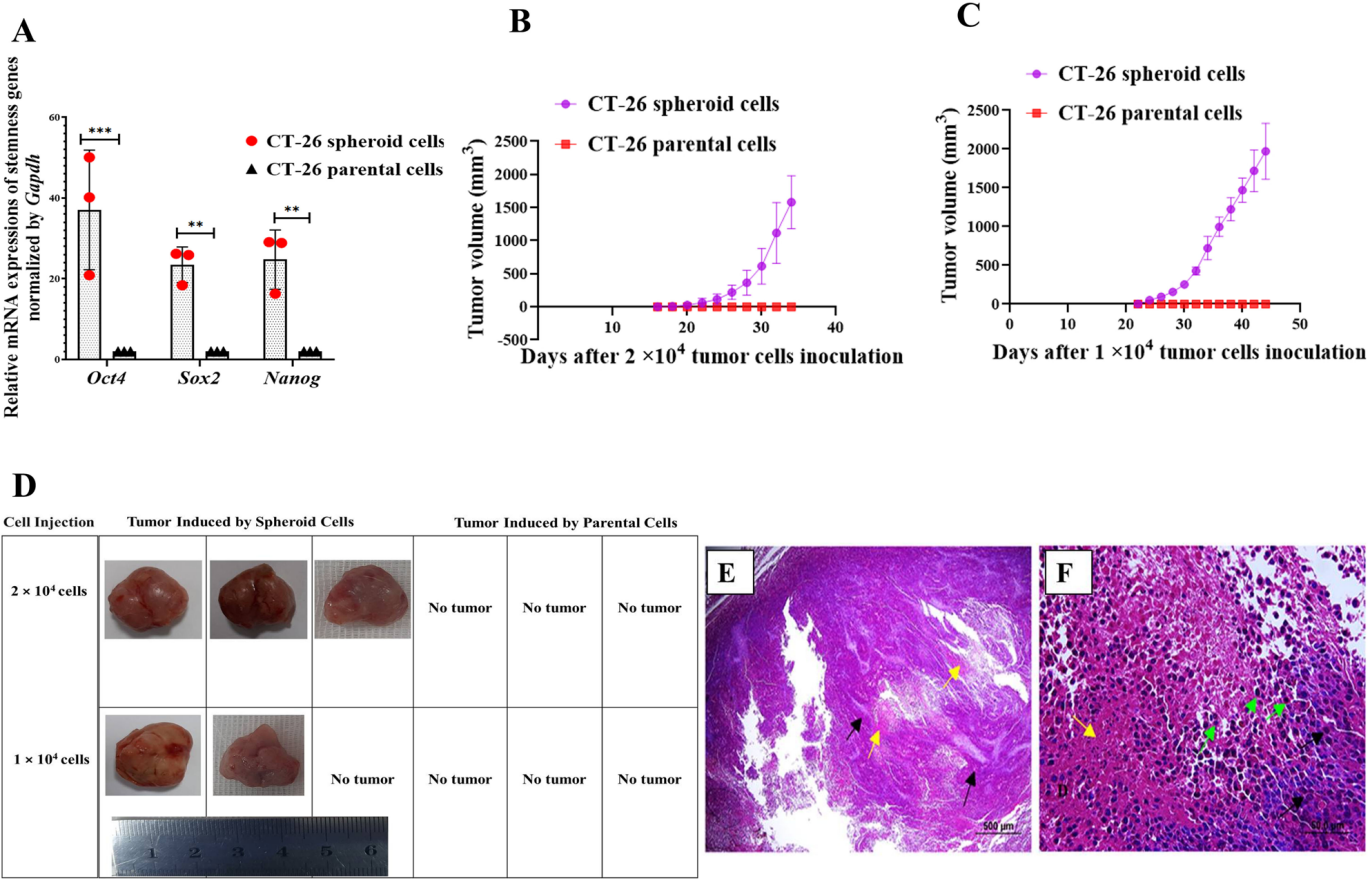
CT-26-derived spheroid cells showed higher expression of stemness genes and enhanced tumorigenic capacity compared to parental cells. **A**, The expression level of stemness genes (*Oct4*,* Sox2*, and *Nanog*) was significantly increased in CT-26 spheroid cells compared to CT-26 parental cells by RT-qPCR (***P = 0.0002, **P = 0.0081, **P = 0.0054, respectively). Scatter plots indicate individual biological replicates and are overlaid with bar charts representing mean expression levels. **B**, 2 × 10^4^, and **C**, 1 × 10^4^ CT-26 spheroid and parental cells were injected into the opposite side of the same mouse. Tumor growth and mean tumor volume were monitored. Tumors appeared on mean days of 19.3 (2 × 10^4^ cells) and 22 (1 × 10^4^ cells) after injection of spheroid cells, whereas injecting an equal number of parental cells into the opposite side of the same mouse did not result in tumor formation (*n* = 3). **D**, Representative images of tumors induced by 2 × 10^4^ and 1 × 10^4^ CT-26 spheroid cells in BALB/c mice. **E**,and** F**, Tumor tissue derived from spheroid cells consisted of sheaths of ovoid to round cells (black arrow), foci of hemorrhage, necrosis (yellow arrow), and macrophage infiltration (green arrow); magnifications 100×, and 400×, respectively

By analyzing the expression of *Oct4*, *Sox2*, and *Nanog* as master regulators of pluripotency and self-renewal properties using real-time PCR, the enrichment of cancer stem-like cells was confirmed. Spheroids exhibited substantially higher expression levels of stemness genes when compared to similar passages of CT-26 parental cells (CT-26 adherent cultures) (Fig. 2A).

##### Spheroid cells exhibit high tumorigenicity potential compared to parental cells in a syngeneic mouse tumor model

To assess the tumorigenicity of CT-26-spheroid cells compared to parental cells, 2 × 10^4^ and 1 × 10^4^ spheroid cells and parental cells in third passages were subcutaneously injected into the opposite flanks of the immunocompetent syngeneic BALB/c host in two separate groups. Tumor formation by spheroid cells was consistently observed at both tested cell doses, demonstrating that their enhanced tumorigenicity potential, compared to parental cells, was maintained across different cell dilutions. Although spheroid cells generated tumors on a mean of 19.3 (2 × 10^4^ cells) and 22 (1 × 10^4^ cells) days after injection, equal numbers of parental cells injected into the opposite side of the same mouse failed to generate tumors (Fig. 2B and C, and 2D). Histologic examination of the lesions induced by spheroid cells revealed an infiltration of ovoid to round neoplastic cells with large vesicular and hyperchromatic nuclei and multiple prominent nucleoli. Neoplastic cells revealed a solid pattern, and the mitotic index (MI) was 2.5%. Multiple focal necrotic and hemorrhagic areas were infiltrated by leukocytes and neoplastic cells at the periphery (Fig. 2E and F).

Collectively, these populations were identified as mouse colorectal cancer stem-like cells and were subsequently utilized in the subsequent assays to investigate their capacity to trigger anti-tumor immune responses in the immunocompetent mice.

### Phase II: efficacy of a therapeutic CSC lysate-based vaccine in tumor-bearing BALB/c mice

**Fig. 3:**
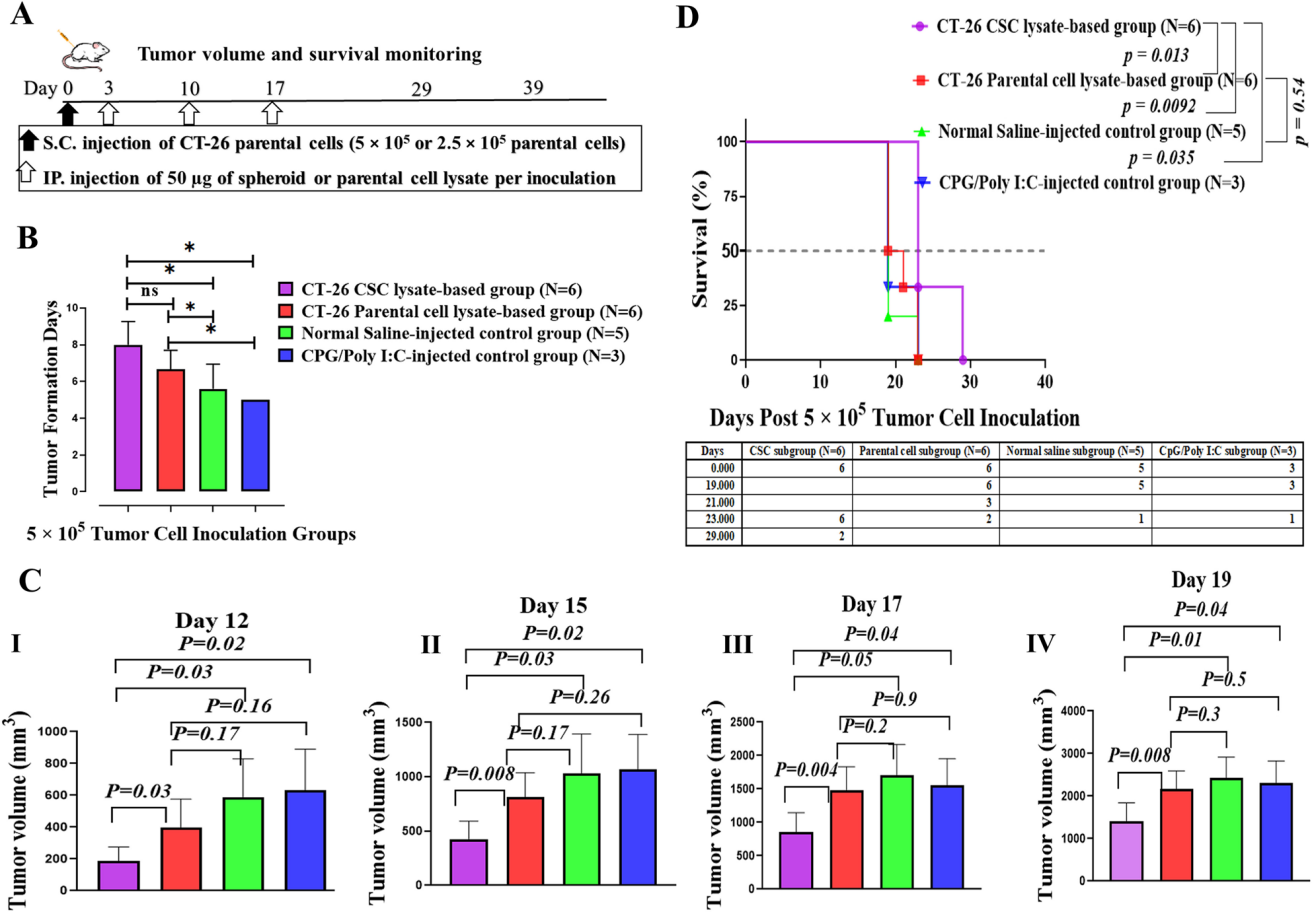
Therapeutic CSC lysate-based vaccines delay tumor formation, reduce tumor growth rates, and enhance the life span of tumor-bearing mice in Group A.** A**,Schematic protocol of BALB/c mice immunization with CT-26 spheroid or parental cell-lysate-based vaccines. BALB/c mice were challenged with CT-26 parental cells, injecting 5 × 10^5^ (Group A) and 2.5 × 10^5^ (Group B) CT-26 parental cells, and, after three days, received intraperitoneal injections of CT-26-CSC lysate, parental cell lysate, CpG/Poly (I: C), and normal saline three times at one-week intervals. **B**, Tumor formation was significantly delayed in the CT-26 CSC lysate-based group compared to the control groups. **C **(**I**,** II**,** III**,** IV**), Comparative analysis of tumor growth was performed after tumor induction in vaccinated mice. The results indicated a significantly reduced tumor volume in the CT-26 CSC lysate-based group compared to other groups on all days. **D**, Kaplan–Meier survival analysis, with comparison by a two-sided log-rank test, indicated that mice immunized with CT-26 CSC lysate survived longer than those in other groups, with median survival times of 23, 20, 19, and 19 days for the CT-26-CSC lysate, CT-26 parental lysate, CpG/Poly (I: C), and saline groups, respectively

The colorectal mouse model was obtained by injecting 5 × 10^5^ (Group A) and 2.5 × 10^5^ (Group B) CT-26 parental cells under the flank skin of two groups of BALB/c mice. Mice were injected intraperitoneally on days 3, 10, and 17 with the CT-26 CSC-lysate, the CT-26 parental cell-lysate, the CpG/Poly (I: C), and normal saline as control groups following tumor induction (Fig. 3A). Subsequent monitoring of tumor volume showed the following results:

#### Delay in tumor formation, reduced tumor growth, and enhanced survival following the administration of therapeutic CSC-lysate-based vaccine to group a of tumor-bearing BALB/c mice

In Group A, in which tumor induction was performed by 5 × 10^5^ CT-26 cells, the CT-26 CSC lysate group showed a delay in tumor formation (average 8 days) compared to the CT-26 parental cell lysate-based (average 6.6 days) and control groups with average tumor generation of 5 and 5.6 days in the CpG/Poly (I: C)-injected control group and normal saline-injected control group, respectively. The delay in tumor formation observed in the CT-26 CSC lysate-based group was statistically significant compared to the control groups. However, there was no statistically significant difference between the tumor formation delay times of the parental cell lysate-based group compared to the CSC lysate-based vaccinated group (Fig. 3B). The difference between the parental cell lysate-based and control groups was also significant.

The average tumor volume was measured and compared across different groups on each specified day to assess the tumor growth rate. This comparative analysis was conducted up to the 19th day following tumor induction, by which time the largest dimension of a tumor in each group had reached 2 cm. As shown in Fig. 3C I-IV, mice immunized with CT-26 CSC lysate-based vaccines had significantly less average tumor volume compared to the CT-26 parental cell lysate-based and control groups on days 12, 15, 17, and 19 following tumor induction.

For example, on day 19, the average tumor volume was 1418 mm³ for the CT-26 CSC lysate-based group, compared to 2163 mm³ for the CT-26 parental cell lysate-based group. In the control groups, the average tumor volume was 2307 mm³ for the CpG/Poly (I: C) group and 2436 mm³ for the normal saline-injected group (Fig. 3C, IV). On this day, the differences between the CT-26 CSC lysate-based group, compared to the CT-26 parental cell lysate, CpG/Poly (I: C)-injected, and normal saline-injected groups, were statistically significant, with *p-values* of *0.008*,* 0.01*,* and 0.04*, respectively (Fig. 3C, IV). In addition, no statistically significant difference existed between mice vaccinated with parental cell lysate and the normal saline-injected group on these days (Fig. 3C, I-IV).

Treatment with CT-26-CSC lysate-based vaccines also prolonged the survival of tumor-bearing mice and exhibited the best outcome relative to other groups (Fig. 3D). The median survival of the CT-26-CSC lysate-based group, the CT-26 parental cell lysate-based group, the CpG/Poly (I: C)-injected control group, and the normal saline-injected control group was 23, 20, 19, and 19 days, respectively. Of note, two mice from the CT-26-CSC lysate-based group could survive 29 days following tumor inoculation.

Results also revealed significant differences between the CT-26-CSC lysate-based group and the CT-26 parental cell lysate-based group (*p* = 0.013), the CpG/Poly (I: C)-injected control group (*p = 0.035*), and the normal saline-injected control group (*p = 0.0092*) (log-rank, Mantel-Cox test) (Fig. 3D). No statistically significant differences existed between mice vaccinated with parental cell lysate and mice injected with normal saline (*p = 0.54*). These results demonstrate that the CSC lysate-based vaccine could improve overall survival against established tumors compared to the parental cell lysate-based vaccine.

#### Delay in tumor formation, reduced tumor growth, and enhanced survival following the administration of therapeutic csc-lysate-based vaccine to group b of tumor-bearing BALB/c mice

**Fig. 4:**
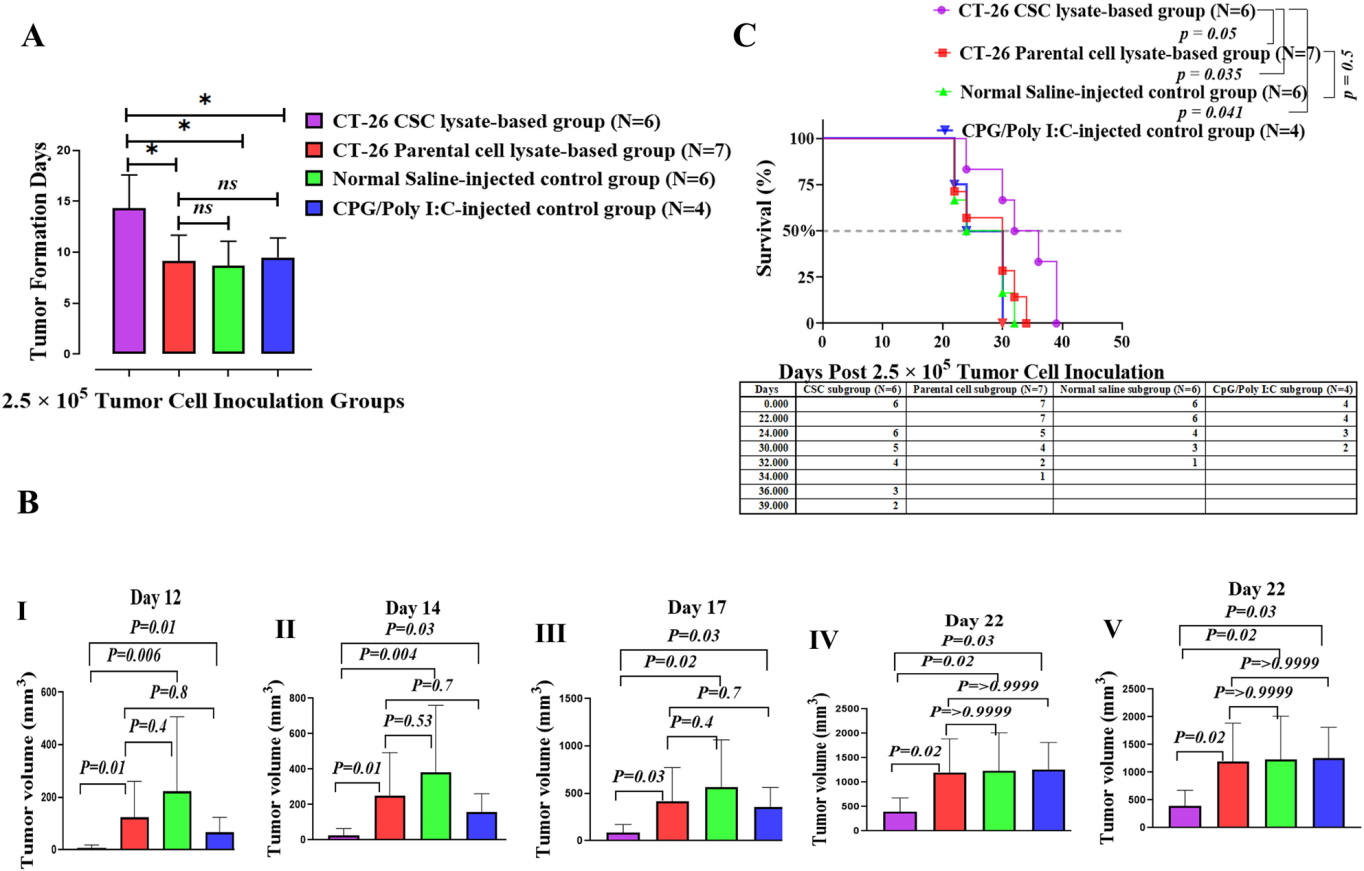
Therapeutic CSC lysate-based vaccines delay tumor formation, reduce tumor growth rate, and enhance the life span of tumor-bearing mice in Group** B**. **A**, Tumor formation was significantly delayed in the CT-26 CSC lysate-based group compared to the other groups. **B (I**,** II**,** III**,** IV**,** V)**, Comparative analysis of tumor growth was performed after tumor induction in vaccinated mice. The results indicated a significantly reduced tumor volume in the CT-26 CSC lysate-based group compared to other groups on all days. **C**, Kaplan–Meier survival analysis, with comparison by a two-sided log-rank test, indicated that mice immunized with CT-26 CSC lysate survived longer than those in other groups, with median survival times of 34, 30, 27, and 27 days for the CT-26-CSC lysate, CT-26 parental lysate, CpG/Poly (I: C), and saline groups, respectively

In Group B, where tumor induction was performed by 2.5 × 10^5^ CT-26 cells, the CT-26 CSC lysate-based group showed a delay in tumor formation (average 14.3 days) compared to the other groups, with averages of 9.14, 9.5, and 8.6 days for the CT-26 parental cell lysate-based group, CpG/Poly (I: C)-injected control group, and normal saline-injected control group, respectively (Fig. 4A). The differences in tumor formation times for the CT-26 CSC lysate-based group, compared to the CT-26 parental cell lysate-based and control groups, were statistically significant, with *p-values* of *0.014*,* 0.04*, and *0.015*, respectively (Fig. 4A). Additionally, there was no statistically significant difference between the tumor formation times of the parental cell lysate-based and control groups. Moreover, tumors in the CT-26 CSC lysate-based group exhibited a significantly slower growth rate compared to the CT-26 parental cell lysate-based and control groups on days 12, 14, 17, 19, and 22 after tumor induction (Fig. 4B, I-V). For example, on day 22, the average tumor volume was 391 mm³ for the CT-26 CSC lysate-based group, compared to 1200 mm³ for the CT-26 parental cell lysate-based group. In the control groups, the average tumor volume was 1251 mm³ for the CpG/Poly (I: C) group and 1231 mm³ for the normal saline-injected group (Fig. 4B, V). The differences in this day for the CT-26 CSC lysate-based group, compared to the CT-26 parental cell lysate, CpG/Poly (I: C)-injected, and normal saline-injected groups, were statistically significant, with *p-values* of *0.02*,* 0.03*, and *0.02*, respectively (Fig. 4B, V). In addition, no statistically significant differences existed between mice vaccinated with parental cell lysate and control groups in these days (Fig. 4B, I-V).

Treatment with CT-26-CSC lysate-based vaccines also prolonged the survival of tumor-bearing mice compared to CT-26 parental cell lysate-based and control groups (Fig. 4C). The CT-26-CSC lysate-based group, the CT-26 parental cell lysate-based group, the CpG/Poly (I: C)-injected control group, and the normal saline-injected control group had median survivals of 34, 30, 27, and 27 days, respectively. Significantly, two mice from the CT-26 CSC lysate-based group survived for 39 days following tumor inoculation.

Indeed, the results revealed significant differences between the CT-26-CSC lysate-based group and parental cell lysate (*p* = 0.05), CpG/Poly (I: C)-injected (*p* = 0.041), and the normal saline-injected groups (*p* = 0.035) (log-rank, Mantel-Cox test) (Fig. 4C).

### Phase III: tumor tissue analysis and immune response evaluation

#### Histological analysis of tumor tissues

**Fig. 5:**
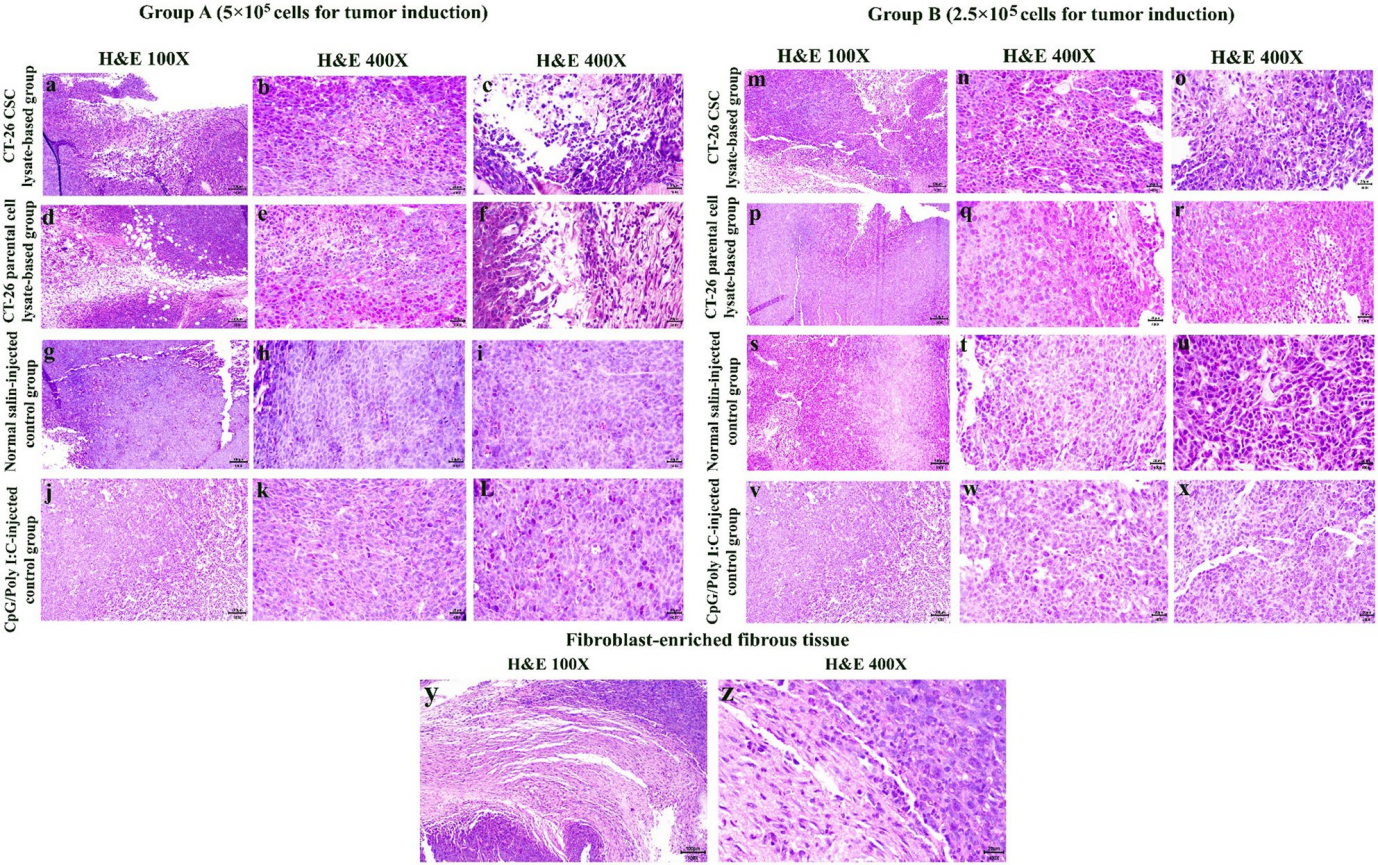
Representative H&E-stained sections of tumor tissues from CT-26 tumor-bearing female BALB/c mice subjected to different vaccination subgroups. Group A (5 × 10⁵ cells for induction): **(a-c) **CT-26 CSC lysate-based subgroup: demonstrating uniform cellular morphology, low mitotic index (MI = 1.56%), extensive necrosis, prominent inflammatory cell infiltration at the central tumor and necrotic margins, and fibroblast-enriched fibrous tissue within the parenchyma (y, z). (d-f) CT-26 parental cell lysate-based subgroup: exhibiting uniform cellular morphology, low mitotic index (MI = 2.19%), significant lymphocytic infiltration, and fibrosis. (g-i) Normal saline-injected control subgroup: characterized by high cellular density, abundant mitotic activity (MI = 5.81%), minimal inflammatory cell infiltration, and limited necrosis. (j-l) CpG/Poly (I: C) control subgroup: displaying very high cellular density, pronounced mitotic activity (MI = 6.60%), mild necrosis, and minimal inflammatory cell infiltration. Group B (2.5 × 10⁵ cells for induction): (m-o) CT-26 CSC lysate-based subgroup: showing uniform cellular morphology, low mitotic index (MI = 2.7%), central necrosis, inflammatory cell infiltration, and fibroblast-enriched fibrous tissue within the parenchyma (y, z). (p-r) CT-26 parental cell lysate-based subgroup: exhibiting high mitotic activity (MI = 4.17%), limited necrosis, mild inflammatory cell infiltration, and fibrous tissue surrounding the tumor. (s-u) Normal saline-injected control subgroup: demonstrating severe cellular pleomorphism, abundant mitotic activity (MI = 8.3%), and invasive cells infiltrating surrounding tissues. (v-x) CpG/Poly (I: C) injected control subgroup: showing high mitotic activity (MI = 5.8%), mild necrosis, and localized invasive cells at the tumor margins (scale bar: a, d, g, j, m, p, s, v, y: 100 μm, b, e, h, k, c, f, i, l, n, q, t, w, o, r, u, x, z: 20 μm)

Histological evaluation of colorectal tumors in murine models subjected to various treatments revealed distinct tumor characteristics—including mitotic activity, inflammatory responses, necrosis, and fibrotic changes—among the experimental and control subgroups, as shown in Figs. 5 (a–z), S1, and Table 2. In Group A (5 × 10^5^) cells for tumor induction, the CT-26 CSC lysate-based group exhibited a low mitotic index (MI = 1.56 ± 0.3 per 10 HPF) and uniform cell pleomorphism. Inflammatory cells, including lymphocytes, neutrophils, and macrophages, infiltrated the tumor center and necrotic margins. Substantial necrosis was evident, accounting for 33.65 ± 6.8% of the tumor area, accompanied by apoptotic bodies, fatty infiltration, and pronounced fibrotic changes (fibrosis score 2.5 ± 0.5). The CT-26 parental cell lysate-based group showed a similarly low mitotic index (MI = 2.19 per 10 HPF) and uniform cell morphology, with central necrosis comprising 28.19 ± 6.7% of the tumor area, along with apoptotic bodies, lymphocytic infiltration, and fatty infiltration. Fibrous tissue and inflammatory cells were prominent at the tumor margins, with a moderate fibrosis score (1.8 ± 0.4). In contrast, the normal saline-injected group demonstrated high cell density and mitotic activity (MI = 5.81 ± 0.6 per 10 HPF) with minimal inflammatory infiltration. Necrosis was limited (9.64 ± 2.2%), and fibrosis was minimal (score 0.1 ± 0.1), suggesting a highly proliferative and potentially aggressive tumor phenotype. The CpG/Poly I: C-injected group exhibited high cell density and a marked mitotic index (MI = 6.60 per 10 HPF), with minimal inflammatory cell infiltration, only mild necrosis (10.96 ± 1.0%), and negligible fibrosis (score 0.3 ± 0.6), indicating limited tissue remodeling within the tumor. In Group B (2.5 × 10^5^) cells for tumor induction, the CT-26 CSC lysate-based group exhibited a low mitotic index (MI = 2.7 ± 0.5 per 10 HPF) and limited cellular pleomorphism. Substantial central necrosis was observed, accounting for 32.89 ± 10.0% of the tumor area, along with apoptotic bodies, fatty infiltration, and infiltration of inflammatory cells, including mononuclear cells and neutrophils, suggesting active tumor–immune interactions and advanced necrotic progression. Prominent fibrous tissue was detected within the tumor center, corresponding to a fibrosis score of 2.5 ± 0.5. The CT-26 parental cell lysate-based group displayed a higher mitotic index (MI = 4.17 per 10 HPF) with moderate necrosis (26.94 ± 5.7%). Fibrous tissue partially surrounded the tumor (fibrosis score 1.5 ± 0.7), and mild inflammatory cell infiltration was observed at the tumor margins. The normal saline-injected group showed tumor cells with heterochromatic nuclei, prominent nucleoli, and severe pleomorphism, accompanied by abundant mitotic activity (MI = 8.3 ± 0.9 per 10 HPF). Invasive tumor cells infiltrating adjacent tissues, including subcutaneous muscle, were frequently observed. Necrosis (8.91 ± 1.7%) and fibrosis (score 0.3 ± 0.5) were minimal, indicating highly aggressive tumor behavior with metastatic potential. The CpG/Poly I: C-injected group exhibited a high mitotic index (MI = 5.8 per 10 HPF) with mild necrosis (10.16 ± 2.7%) and minimal fibrosis (score 0.5 ± 0.6). An absence of inflammatory cells in the tumor center and the presence of single invasive cells at the tumor margins suggested localized invasion associated with a low inflammatory response.

Collectively, across both inoculation groups, the CSC lysate-based vaccination consistently produced the lowest mitotic indices, highest necrotic areas, and highest fibrosis scores relative to controls, reflecting superior anti-tumor immune engagement and tumor microenvironment remodeling compared with parental lysate, CpG/Poly (I: C), and normal saline-injected control groups (*p* < 0.05).

**Table 2:** Histopathological analysis of tumor tissues in different vaccination subgroups of Groups A and B

Subgroups	Inflammatory Responses	Mitotic Index (MI)	Cellular Pleomorphism	Necrosis	Fibrosis	Single Invasive Cells at Margins	Others
**Group A (5 × 10⁵ cells for tumor induction)**							
CT-26 CSC lysate-based subgroup	Moderate inflammation (Neutrophils and lymphocytes at the tumor center; lymphocytes, plasma cells, and neutrophils at the margins)	Low (MI = 1.56%)	Uniform	33.65 ± 6.8%	2.5 ± 0.5	No	Apoptotic bodies, fatty infiltration
CT-26 parental cell lysate-based subgroup	Moderate inflammation (lymphocytes and neutrophils at the tumor center)	Low (MI = 2.19%)	Uniform	28.19 ± 6.7%	1.8 ± 0.4	No	Apoptotic bodies, fatty infiltration
Normal saline-injected subgroup	Minimal inflammation (few neutrophils and lymphocytes at the margins)	High (MI = 5.81%)	Severe	9.64 ± 2.2%	0.1 ± 0.1	Yes	None
CpG/Poly (I: C)-injected subgroup	Minimal inflammation (few neutrophils and lymphocytes at the margins)	High (MI = 6.60%)	Severe	10.96 ± 1.0%	0.3 ± 0.6	Yes	None
**Group B (2.5 × 10⁵ cells for tumor induction)**							
CT-26 CSC lysate-based subgroup	Moderate inflammation (Neutrophils and lymphocytes at the tumor center and margins)	Low (MI = 2.7%)	Limited cellular pleomorphism	32.89 ± 10.0	2.5 ± 0.5	No	Apoptotic bodies, fatty infiltration
CT-26 parental cell lysate-based subgroup	Minimal inflammation (Neutrophils and lymphocytes at the tumor margins)	High (MI = 4.17%)	Severe	26.94 ± 5.7%	1.5 ± 0.7	No	None
Normal saline-injected subgroup	Minimal inflammation (few neutrophils and lymphocytes at the margins)	High (MI = 8.3%)	Severe	8.91 ± 1.7%	0.3 ± 0.5	Yes	None
CpG/Poly (I: C)-injected subgroup	Minimal inflammation (few neutrophils and lymphocytes at the margins)	High (MI = 5.8%)	Severe	10.16 ± 2.7%	0.5 ± 0.6	Yes	None

#### Therapeutic CT-26 cell lysate-based vaccine elicited the production of anti-csc and anti-parental cell antibodies in immunized mice

**Fig. 6:**
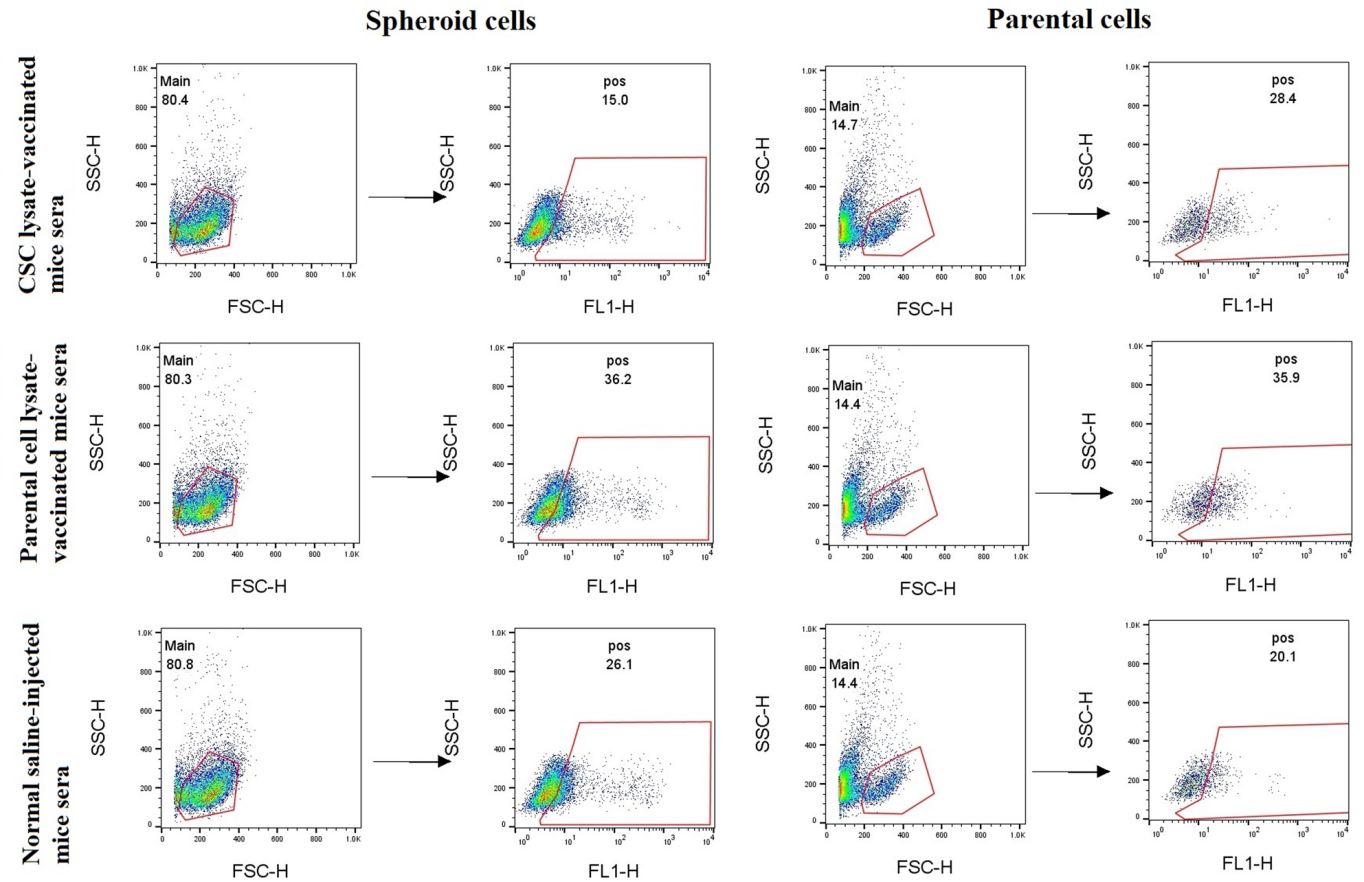
Flow cytometry showed the immunoreactivity of vaccinated mice with CT-26 spheroid and parental cell populations in Group A (5 × 10^5^ cells for tumor induction). Immune sera from CT-26 CSC lysate-vaccinated mice recognized 15% spheroid cells and 28.4% parental cells, respectively (first panel). Also, immune sera from CT-26 parental cell lysate-vaccinated mice recognized 36.2% spheroid cells and 35.9% parental cells, respectively (second panel). Data were obtained using pooled sera from each experimental group and are presented as representative group-level measurements

**Fig. 7:**
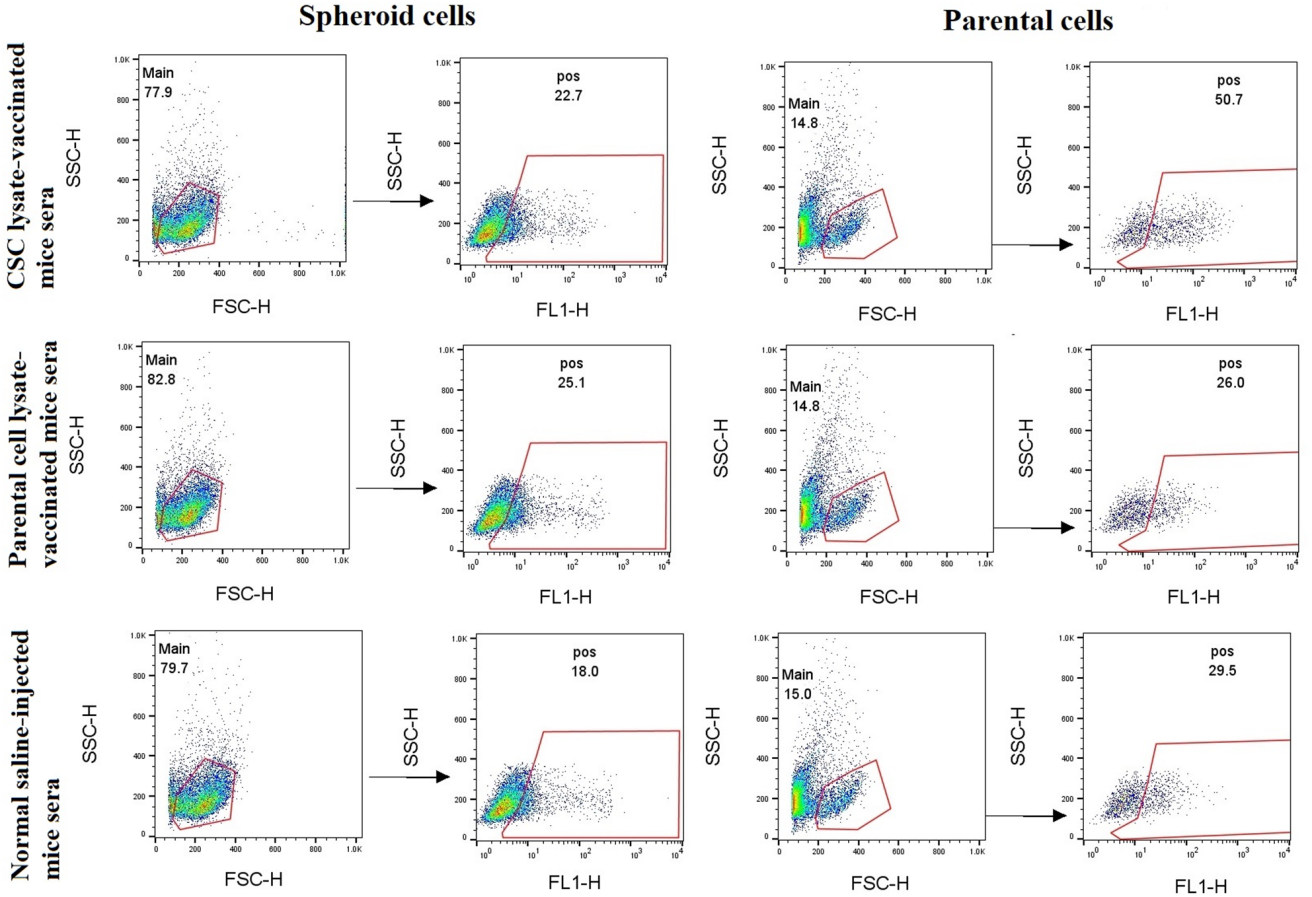
Flow cytometry showed the immunoreactivity of vaccinated mice sera with CT-26 spheroid and parental cell populations in Group B (2.5 × 10^5^ cells for tumor induction). Immune sera from CT-26 CSC lysate-vaccinated mice recognized 22.7% spheroid cells and 50.7% parental cells, respectively (first panel). Also, immune sera from CT-26 parental cell lysate-vaccinated mice recognized 25.1% spheroid cells and 26% parental cells, respectively (second panel). Data were generated using pooled sera from each experimental group and are presented as representative group-level measurements

To provide experimental evidence that therapeutic CSC lysate-based and parental cell lysate-based vaccines induce anti-CSC and anti-parental cell immunity, we collected sera from vaccinated and control mice (Groups A and B) and analyzed them for antibody production by detecting the reactivity of mice’s sera to CSCs and parental cell populations. Background fluorescence and gating were defined using unstained cells and cells incubated with sera from normal (non-immunized) mice for each cell population (Figure S2). The immunoreactivity of pooled mice sera from the vaccinated groups with lysates of CSC and parental cells in Groups A and B is shown in Figs. 6 and 7. In Group A (5 × 10^5^ cells for tumor induction), flow cytometry analysis indicated that 15% of spheroid cells were positive compared to parental cells (28.4%) when serum from CT-26 CSC lysate-vaccinated mice was used. Also, immune sera from CT-26 parental cell lysate-vaccinated mice showed 36.2% and 35.9% reactivity against spheroid cells and parental cells (Fig. 6). Furthermore, sera from normal saline-injected mice recognized the spheroid and parental cells with 26.1% and 20.1%, respectively, indicating intrinsic immune response activation against the tumor after tumor induction. The results indicate that CT-26 parental cell lysate-vaccinated mice recognize spheroid cells and parental cells more than the sera from the CT-26 CSC lysate-based vaccinated group (Fig. 6).

In Group B (2.5 × 10^5^ cells for tumor induction), flow cytometry analysis demonstrated CT-26 CSC lysate-vaccinated mice recognize the spheroid and parental cells with 22.7% and 50.7%, respectively. Additionally, immune sera from CT-26 parental cell lysate-vaccinated mice recognized spheroid cells and parental cells to approximately the same extent (25.1% and 26%, respectively) (Fig. 7). Furthermore, immunostaining with sera from normal saline-injected mice revealed that 18% of spheroid cells were positive compared to parental cells with 29.5% positive cells. Collectively, CSC lysate-based vaccinated mice recognized parental cells more than the sera from the CT-26 parental cell lysate-based vaccinated group (50.7% and 26%, respectively) (Fig. 7).

**Fig. 8:**
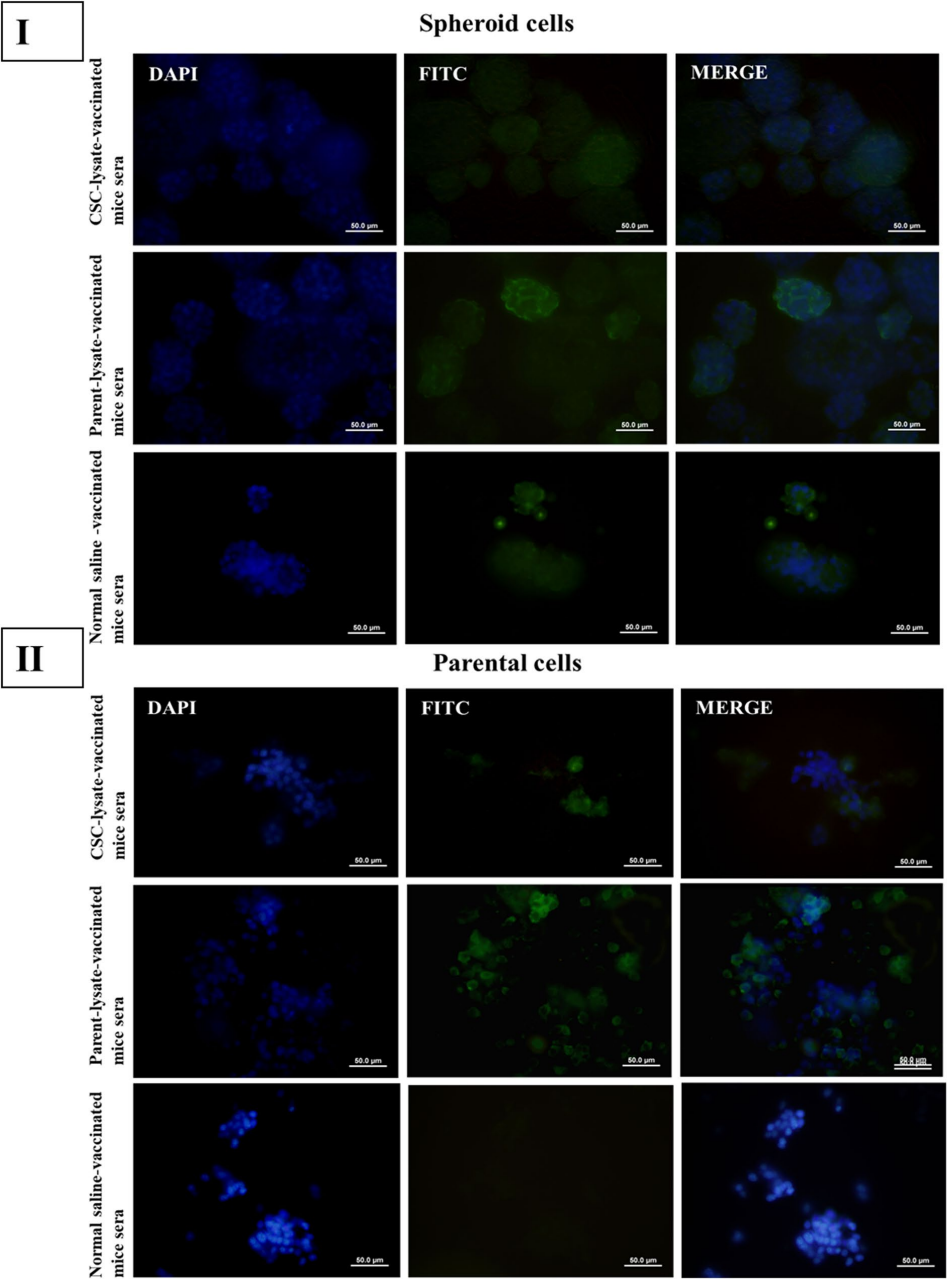
Immunization with CT-26 CSC and parental cell lysate induced the production of antibodies against spheroid and parental cell antigens in Group A (5 × 10^5^ cells for tumor induction). **I**, Immunofluorescent staining of CSC lysate-vaccinated, parental cell lysate-vaccinated, and normal saline-injected mice showed immunoreactivity in fixed CT-26 spheroids. Immunofluorescent staining of parental cell-lysate-induced antibodies displayed more reactivity against CT-26 spheres. **II**, Immunofluorescent staining of CT-26 parental cells with sera from parental cell lysate, CSC lysate-based mice, and normal saline-injected mice revealed parental cell lysate-induced antibodies were highly reactive against parental cells. Antibody reactivity to spheroids and parental cells was visualized using FITC-conjugated anti-mouse IgG antibody and a merged image of DAPI and FITC. Immunofluorescence images are presented as representative qualitative visualizations of antibody reactivity

**Fig. 9:**
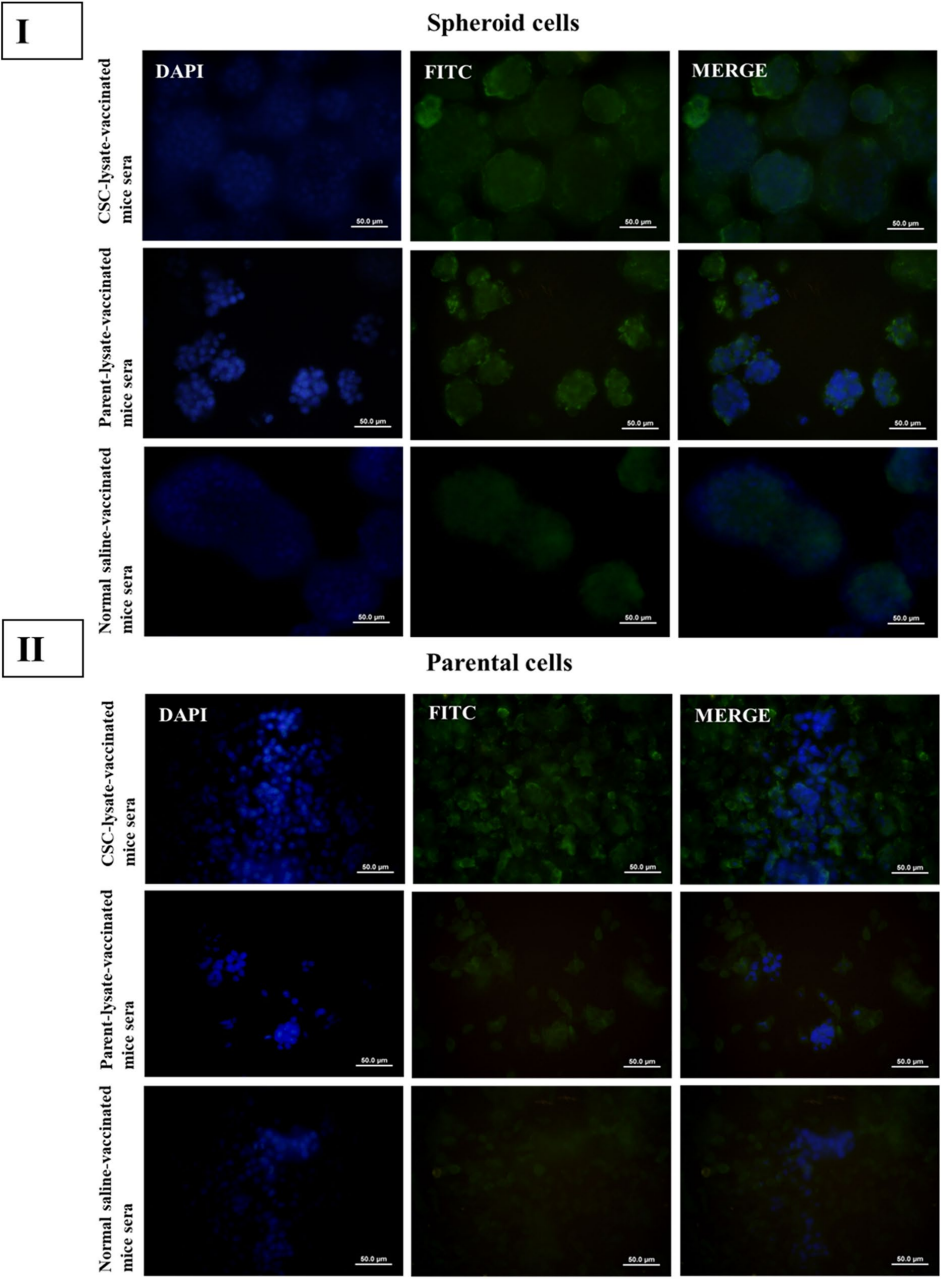
Immunization with CT-26 CSC and parental cell lysate induced the production of antibodies against spheroid and parental cell antigens in Group B (2.5 × 10^5^ cells for tumor induction).** I**, Immunofluorescent staining of CSC lysate-vaccinated, parental cell lysate-vaccinated, and normal saline-injected mice showed immunoreactivity in fixed CT-26 spheroids. Immunofluorescent staining of parental cell-lysate-induced antibodies displayed more reactivity against CT-26 spheres. **II**, Immunofluorescent staining of CT-26 parental cells with sera from parental cell lysate, CSC lysate-based mice, and normal saline-injected mice revealed that parental cell lysate-induced antibodies were highly reactive against parental cells. Antibody reactivity to spheroids and parental cells was visualized using a FITC-conjugated anti-mouse IgG antibody and a merged image of DAPI and FITC. Immunofluorescence images are presented as representative qualitative visualizations of antibody reactivity

The IF test results revealed that the serum reactions of the CT-26 CSC lysate and parental cell lysate-based vaccine groups were similar to those observed in the flow cytometry test (Figs. 8 and 9).

## Discussion

Tumor eradication requires eliminating CSCs, a tiny proportion of stem cell-like cells in tumor mass that have a key role in tumor development, metastasis, recurrence, and heterogeneity due to their resistance to current therapies [34–36]. CSC populations may lack these differentiated tumor antigens; therefore, these immunotherapeutic strategies may not target them [37]. In addition, CSCs can possess several immune evasion strategies; through genetic and non-genetic changes to establish unique neoantigens, reduce the expression of tumor antigens, increase immune-mediated cytotoxicity tolerance, and create a protective immunosuppressive tumor microenvironment (TME) [38]. Hence, an unmet need exists to develop innovative treatment approaches to combat CSCs.

Current immunotherapy strategies for targeting CSCs include antibody-based therapies, immune checkpoint inhibitors, chimeric antigen receptor T cells (CAR-T cells), and whole CSC-based vaccines [22, 39]. Among these approaches, whole CSC-based vaccines are particularly promising due to their theoretical advantages over conventional therapies. Specifically, targeting multiple epitopes simultaneously provides substantial benefits compared to focusing on a single epitope [40, 41]. This multi-epitope targeting approach is believed to significantly reduce the risk of immune evasion by malignant cells, thereby enhancing therapeutic efficacy [42, 43].

No cancer vaccine targeting CSCs has yet been approved, although many are undergoing clinical trials [22]. The antigenic profile of CSCs seems to be sophisticated. Moreover, identifying and characterizing molecules with immunogenic potential complicates this scenario [24]. Therefore, at first, candidate neoantigens must be identified and subsequently verified for their capability for effective major histocompatibility complex (MHC) molecular presentation (Dendritic cells (DC) and B cells) and recognition by T-cell receptors (TCRs) [44]. Consequently, substantial effort is still warranted to access the potential immunogenicity of neoantigen candidates. Strategies that target whole CSCs may be an effective way to address this challenge. In addition, humoral responses can be induced against CSC antigens following whole CSC-based vaccination. It seems that this approach could be effective in the identification of CSC antigens [22, 29].

From a therapeutic point of view, most studies have shown delayed tumor generation, decreased tumor volume, and metastasis, and an increased survival rate in animal cancer models following whole CSC lysate-based vaccination as an immunotherapeutic approach [22, 45]. CSC-vaccinated hosts additionally exhibit elevated levels of interleukin 12 (IL-12) [46], interferon-gamma (IFN-γ) [47], and IgG levels [48]. Moreover, they showed enhanced cytotoxicity of both natural killer cells (NK cells) [49] and cytotoxic T cells (CTLs) [47], lower T regulatory lymphocyte number (Treg) [50], and low level of transforming growth factor-β (TGF-β) [51]. Considering the therapeutic effects and the induction of antibody responses following whole CSC-based vaccination, it can be concluded that this approach led to the identification of immunogenic CSC-specific antigens with the potential to be used in targeted therapy against cancer; for instance, receptor tyrosine kinase-like orphan receptor 1 (ROR1) is an embryonic protein that functions as a receptor for Wnt5a and is abnormally expressed in cancer stem cells (CSCs). ROR1 is overexpressed on malignant B cells in the majority of chronic lymphocytic leukemia (CLL) patients [52, 53]. In vivo studies show that cirmtuzumab, an anti-ROR1 monoclonal antibody, suppresses stem cell-associated gene expression in CLL. Clinical trials further demonstrate that cirmtuzumab effectively downregulates the ROR1 signaling pathway in these patients [52, 53].

Our previous study evaluating the prophylactic efficacy of CT-26 CSC lysate-based vaccination showed inhibition of tumor growth and the extension of tumor-bearing mice’s survival in BALB/c mice [30]. Cancer vaccines can serve as preventive measures in high-risk populations, known as prophylactic vaccines, and as treatment options for individuals already diagnosed with cancer, referred to as therapeutic vaccines [54]. Therapeutic cancer vaccines are, at present, more closely aligned with our everyday lives than preventive cancer vaccines. Therefore, anti-tumor therapeutic vaccines are the subject of significant research and development.

The present study was designed to gain more profound insight into the therapeutic efficacy of a CSC lysate-based vaccine on tumor development and mouse survival using the CT-26 syngeneic colorectal carcinoma model.

To that end, spheroid cells derived from the CT-26 parental cells, as a CSC-enriched population, showed higher tumorigenicity potential and an elevated level of stemness gene expression compared to their parental counterparts. The therapeutic vaccination with CSC-lysate was administered in two distinct settings, involving a high and low number of CT-26 parental cells (5 × 10^5^ and 2.5 × 10^5^ cells, respectively) for tumor induction. Some studies have shown that the inoculated cell density of tumor cells is a fundamental determinant of tumor latency, the growth dynamics and metastatic potential of the cells, tumor-infiltrating leukocyte populations, and response to immune checkpoint blockade (ICB) therapy [55, 56]. Therefore, to investigate the relationship between the therapeutic effects of CSC vaccination and the number of induced tumor cells, two distinct syngeneic colorectal carcinoma models were generated by the induction of tumor using 5 × 10^5^ and 2.5 × 10^5^ CT-26 cells.

This study showed that CSC-lysate vaccination delayed tumor formation in both groups. In addition, following CSC-based vaccination, tumor growth was significantly reduced compared to the parental cell lysate and control groups for consecutive days in groups A and B for the initial 19- and 22 days following tumor induction, respectively. In addition, no statistically significant differences existed between mice vaccinated with parental cell lysate and the normal saline-injected group during these days. Our study’s findings are consistent with previous research on whole CSC-based vaccination in various cancer mouse models, including those by Liu, Sun, and Sakamoto et al. [46, 57, 58]. Our findings suggest that this vaccine may be more effective in smaller tumors, as its inhibitory effect on tumor growth diminishes with increasing tumor size. These results should be further examined and replicated in studies with larger sample sizes.

Notably, CSC lysate vaccination significantly increased the survival rate of tumor-bearing mice compared to other groups in both Group A and Group B. No macroscopic abnormalities or side effects were observed in the vaccinated mice. In this study, tumor volume was the key factor determining the survival rate.

To understand why CSC immunization effectively produces these outcomes, we evaluated the histopathological and immunological responses in different subgroups. The results showed that in Group A (5 × 10^5^ cells for tumor induction), those vaccinated with CSC and parental cell lysates had significant inflammation at the tumor center, with low mitotic activity, high necrosis, and prominent fibrosis. In contrast, the subgroups treated with normal saline and adjuvants showed minimal inflammation, high mitotic activity, and invasive cells, indicating aggressive tumor behavior. In Group B (2.5 × 10⁵ cells for tumor induction), the CSC lysate subgroup exhibited similar inflammation, low mitotic activity, high necrosis, and fibrosis, while the parental cell lysate subgroup showed minimal inflammation and high mitotic activity. Control subgroups in Group B also displayed minimal inflammation, high mitotic activity, invasive cells, and aggressive tumor characteristics.

In this study, the CSC lysate-based subgroup showed significant infiltration of inflammatory cells, indicating immune system activation. Vaccination led to the recruitment of these cells to the tumor center, reducing tumor cell proliferation. The inflammatory cells were also associated with increased necrosis and fibrosis around the tumor site. This phenomenon has been previously reported; for example, cytotoxic T cells (CD8⁺) not only induce apoptosis in cancer cells but can also cause necrosis under severe cellular damage. This dual action is supported by releasing inflammatory cytokines like Tumor necrosis factor (TNF-α) and IFN-γ [59, 60]. The necrosis may further increase inflammation and tissue damage in tumor regions, enhancing the immune response and boosting the vaccine’s therapeutic effect [61]. Fibrosis is observed in tumor regions, where tissue repair leads to increased collagen production by fibroblasts [62, 63]. This fibrosis stiffens the tumor area, limiting tumor growth and spread [64, 65].

Furthermore, in Group A (5 × 10^5^ cells for tumor induction), no significant differences were observed between CSC and parental cell lysates in immune response. However, in Group B (2.5 × 10⁵ cells for tumor induction), an important difference existed between these two subgroups.

These findings underscore the therapeutic potential of CSC lysate-based vaccines, particularly in Group B, where they appear more effective in modulating tumor growth and enhancing immune-mediated responses. This efficacy aligns with the observation that smaller tumors, characterized by a more appropriate ratio of immune cells to tumor cells, facilitate an effective immune response. However, as the larger tumors and the number of tumor cells increase, this ratio decreases, and the immune response may become significantly weakened [66]. Consequently, these findings emphasize the advantage of CSC lysate-based vaccines in smaller tumors, underscoring their potential as an effective therapeutic strategy in early tumor stages.

Moreover, we assessed the humoral immunity induced by the vaccination, evaluating the antibodies against CSCs and parental cells in all groups using flow cytometry and immunofluorescence (IF) tests. In cancer research, flow cytometry and immunofluorescence (IF) tests complement each other. Flow cytometry precisely quantifies and characterizes marker expressions in tumor samples [67, 68], while the IF test reveals the spatial distribution of proteins within cells or tissues [69]. This combination allows researchers to understand the overall expression levels and the specific cellular localization of important molecules, providing a more comprehensive view of tumor biology and microenvironment.

The results showed that in Groups A and B, the immune sera from the CSC-based vaccine group recognized parental cells (28.4% and 50.7%, in Groups A and B, respectively) more than CSCs (15% and 22.7%, in Groups A and B, respectively). Moreover, the immune sera from the parental cell lysate-vaccinated mice recognize spheroid and parental cells to approximately the same extent in Groups A and B. Furthermore, sera from normal saline-injected tumor-bearing mice displayed reactivity against both CSCs and parental cells.

As mentioned, vaccination with CSCs in Groups A and B reduced tumor growth and increased survival in tumor-bearing mice. However, analysis of the humoral immune response showed that serum from CSC-vaccinated mice recognized parental cells more than CSCs. Two possible explanations can be proposed for the above findings:

(1)While CSCs show low immunogenicity [18, 70], they contain antigens with therapeutic functions (if targeted therapy is administered) [71]: Several lines of evidence indicate the low immunogenicity of CSCs, attributed to several factors including reduced expression of tumor antigens, downregulation of major histocompatibility complex class I (MHC-I) molecules and antigen processing machinery (APM), decreased expression of NK ligands and CD47, secretion of immunosuppressive molecules that attenuate the immune response, recruitment of immune-suppressive cells, and facilitation of CSC immune-evasion strategies through signaling pathways such as Notch, Wnt, and Hedgehog [18, 70]. It is important to note that although all immunogens are necessarily classified as antigens, not all antigens possess the characteristic of functioning as immunogens [72]. Considering the therapeutic effects of CSC-based vaccination and the low immunogenicity of CSC antigens, immunization strategies should be employed to boost the immunogenicity of the CSC antigens, including choosing the right adjuvant, altering the lysate preparation steps, altering the route of administration, and increasing the dose of antigen delivered. For example, to increase immunogenicity, in some previous studies, CSCs were transfected with Molecule mucin1 (MUC1) and type I receptor tyrosine kinase-like orphan receptor 1 (ROR1). Following vaccination, protective efficacy was evaluated in a murine model of colorectal cancer and epithelial ovarian cancer, respectively [47, 49].

(2) Both the humoral (B-cells/antibodies) and cell-mediated (T-cells) arms of the adaptive system are necessary to fight against cancer [73]. In the present study, we aimed to examine antibody production against CSC antigens (the humoral arm), whereas the cell-mediated immune response against CSCs or parental cells has not been investigated. The increased survival of tumor-bearing mice following CSC-based vaccination is likely related to T cell-mediated function [73]. Our previous in vitro study showed that CSC-derived lysates and exosomes promote DC maturation and T-cell activation, leading to significant anti-tumor effects in colorectal cancer [74].

In addition, the presented data indicate humoral responses following parental cell-based vaccination, with antibodies recognizing both parental cells and CSCs, as assessed by flow cytometry and IF, suggesting the recognition of similar epitopes.

Notably, our results also showed that the induction of tumors can induce intrinsic immune response activation. This finding agrees with other researchers’ findings [75–78]. Some mechanisms were proposed to explain this observation. Recent studies have found that necroptosis, a regulated form of cell death, in the vicinity of tumor cells might enhance the immune system’s ability to eliminate cancerous cells from the body [78, 79]. In addition, these findings indicate that the dying cells within the tumor can trigger an immunological response, which operates independently of the immune system’s ability to identify markers on cancer cells. Consequently, it is plausible that cellular death may potentially enhance the efficacy of cancer immunotherapy [78].

## Conclusion

Future progress in therapeutic cancer vaccines should focus on identifying immunogenic neoantigens, overcoming immunosuppression, and exploring combination therapies, as immune activation in the therapeutic setting must occur in the presence of an established tumor, a condition that can pose challenges to achieving long-term tumor control. This study’s promising results in delayed tumor formation, increased survival, and immune response stimulation in CSC lysate subgroups highlight the importance of identifying neoantigens using mass spectrometry. While parental cells showed higher immunogenicity, CSCs demonstrated better therapeutic efficacy, suggesting that identifying functional epitopes in CSCs could improve treatment outcomes. Considering the impact of tumor size on vaccination efficacy, administration of the vaccine when the tumor is small or after tumor size reduction by other means is a plausible strategy, which, however, needs more investigation. In summary, CSC lysate-based vaccines can stimulate both cellular and humoral immune responses, potentially complementing existing treatment strategies.

## Limitations

A limitation of the present study is the lack of dedicated functional immune assays to directly demonstrate effector mechanisms. Although antigen-specific antibody production and pronounced immune cell infiltration in tumor tissues supported the involvement of both humoral and cellular immunity, we did not perform functional analyses such as antibody-dependent cytotoxicity assays, immunohistochemical characterization of tumor-infiltrating immune cell subsets (including CD8⁺ cytotoxic and memory T cells), or direct measurements of cytotoxic T-lymphocyte activity. Comprehensive functional and phenotypic immune analyses will therefore be incorporated in future studies to further elucidate the mechanistic basis of the observed antitumor effects.

## Futures studies

Future studies should focus on determining whether CSC-based lysate vaccination induces durable immunological memory and long-term tumor protection.

## Supplementary Information

Supplementary Material 1.

## Data Availability

The data used to support the findings of this study are available from the corresponding author upon reasonable request.

## Abbreviations

CSCs: Cancer stem cells
H&E: Hematoxylin and eosin staining
CRC: Colorectal cancer
CAR-T cells: Chimeric antigen receptor-modified T cells
TAA: Tumor-associated antigens
TSA: Tumor-specific antigens
DMEM/F12: Dulbecco modified Eagle medium/F12
SEM: Scanning electron microscopy imaging
2-ME: 2-mercaptoethanol
CpG oligonucleotide: Phosphorothioate-modified CpG oligodeoxynucleotide
Poly I:C: Polyinosinic-Polycytidylic acid
IF: Immunofluorescence staining
MI: Mitotic index
TME: Tumor microenvironment
MHC-I: Major histocompatibility complex class I
DC: Dendritic cells
TCRs: T-cell receptors
IL-12: Interleukin 12
IFN-γ: Interferon‐gamma
NK cells: Natural killer
CTLs: Cytotoxic T cells
Treg: T regulatory lymphocyte number
TGF-β: Transforming growth factor-β
ROR1: Receptor tyrosine kinase-like orphan receptor 1
CLL: Chronic lymphocytic leukemia
ICB: Immune checkpoint blockade
TNF-α: Tumor necrosis factor
MUC1: Molecule mucin1
