# Supplementary material for: Therapeutic efficacy of cancer stem cell-based vaccine in colorectal murine model: reduced tumor growth and prolonged survival
Source: BMC Cancer. 2026 Mar 29;26:614. doi: 10.1186/s12885-026-15925-3 (PMC13169681; doi:10.1186/s12885-026-15925-3)
Supplement: Supplementary file 1 — Supplementary Material 1. [file 12885_2026_15925_MOESM1_ESM.docx]

**Supplementary Material**

**Table S1. Percentage of reactive CT-26 spheroid and parental cells detected by flow cytometry using pooled sera from vaccinated mice**

| **Group A (5×10^5^ cells for tumor induction)** | | | |
| --- | --- | --- | --- |
|  | **CSC lysate-based vaccinated mice sera**  **(CSC subgroup sera)** | **Parental lysate-based vaccinated mice sera**  **(Parental subgroup sera)** | **Normal saline-injected mice sera**  **(Normal saline subgroup sera)** |
| **CT-26 spheroid cell populations** | **15%** | **36.2%** | **26.1%** |
| **CT-26 Parental cell populations** | **28.4%** | **35.9%** | **20.1%** |
| **Group B (2.5×10^5^ cells for tumor induction)** | | | |
| **CT-26 spheroid cell populations** | **22.7%** | **25.1%** | **18%** |
| **CT-26 Parental cell populations** | **50.7%** | **26%** | **29.5%** |


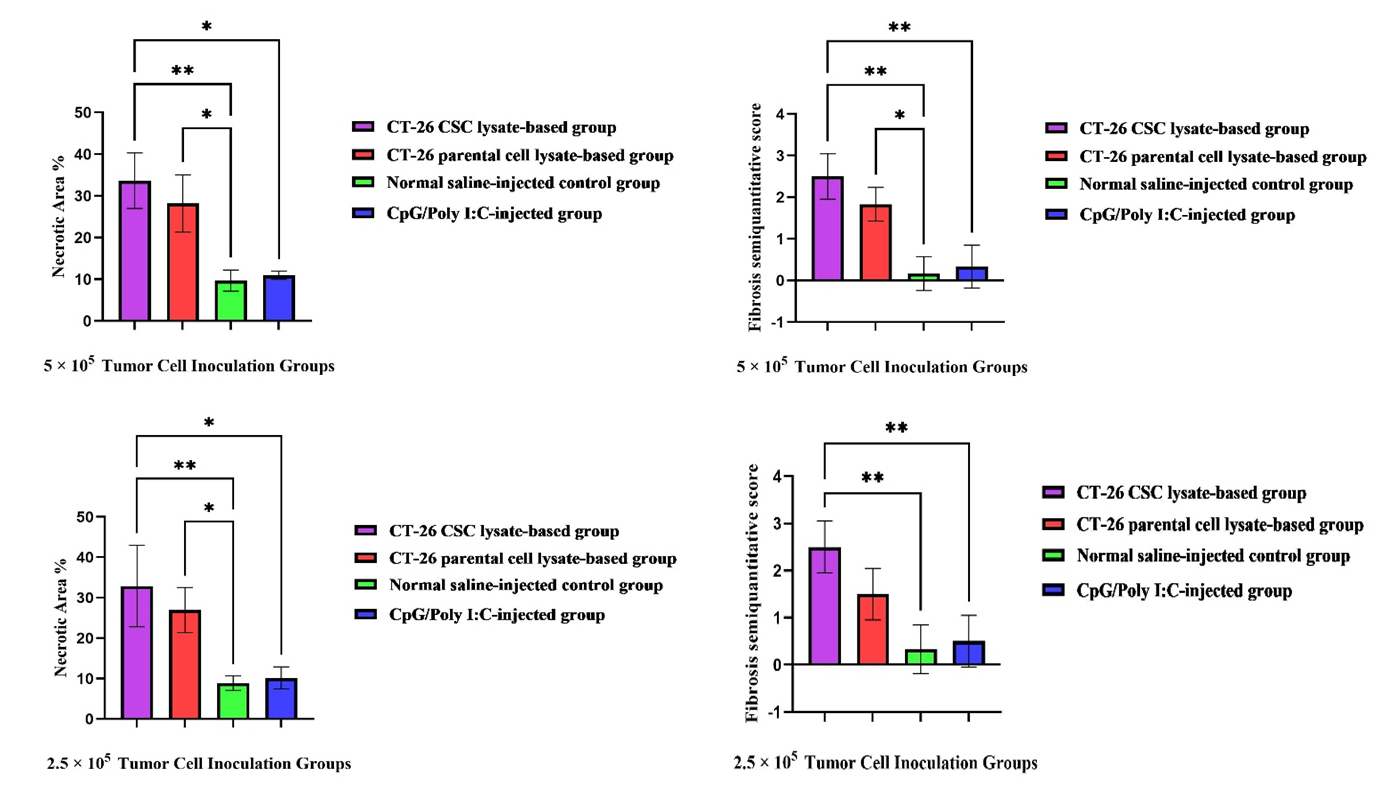


**Fig. S1. Comparative histological assessment of necrosis and fibrosis in experimental subgroups.** Representative histological sections of colorectal tumors from Groups A (5×10⁵ cells for induction) and B (2.5×10⁵ cells for induction) demonstrating the extent of necrosis and fibrotic remodeling across treatment subgroups. In both tumor induction models, the CT-26 CSC lysate–based group exhibited substantial central necrosis (≈33.65±6.8% in Group A; 32.89±10.0% in Group B), accompanied by pronounced fibrotic changes (fibrosis score 2.5±0.5). The CT-26 parental cell lysate–based group showed moderate necrosis (≈28.19±6.7% in Group A; 26.94±5.7% in Group B) with moderate fibrosis (scores 1.8±0.4 and 1.5±0.7, respectively). In contrast, the normal saline–injected and CpG/Poly (I:C)–injected groups displayed limited necrosis (≈8.91–10.96%) and minimal fibrosis (scores 0.1–0.5), consistent with reduced stromal remodeling.


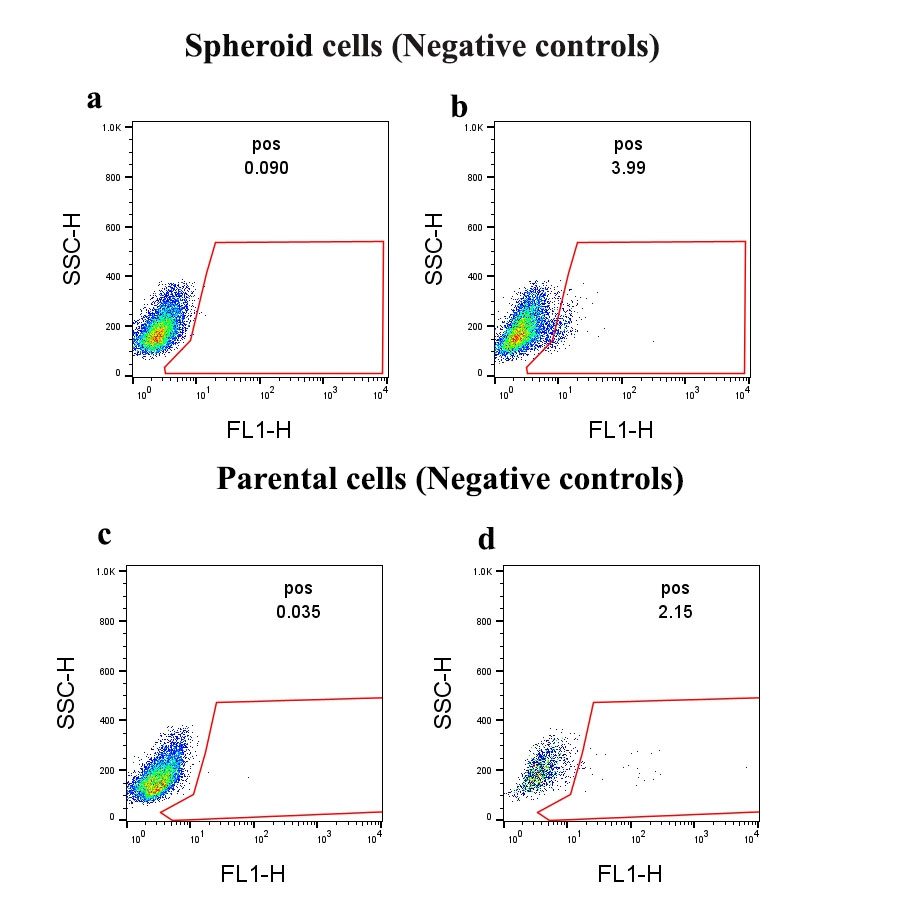


**Fig. S2. Representative flow cytometry profiles of negative controls for spheroid (a,b) and parental (c,d) cells.** Unstained cells and cells stained with non-immunized mouse serum were used to define background fluorescence, set gating thresholds, and exclude nonspecific binding. All samples were analyzed under identical conditions.
